# Short‐wavelength‐sensitive 1 (*SWS1*) opsin gene duplications and parallel visual pigment tuning support ultraviolet communication in damselfishes (Pomacentridae)

**DOI:** 10.1002/ece3.11186

**Published:** 2024-04-16

**Authors:** Sara M. Stieb, Fabio Cortesi, Laurie Mitchell, Luiz Jardim de Queiroz, N. Justin Marshall, Ole Seehausen

**Affiliations:** ^1^ Center for Ecology, Evolution and Biogeochemistry EAWAG Federal Institute of Aquatic Science and Technology Kastanienbaum Switzerland; ^2^ Institute for Ecology and Evolution University of Bern Bern Switzerland; ^3^ Queensland Brain Institute The University of Queensland Brisbane Queensland Australia; ^4^ School of the Environment The University of Queensland Brisbane Australia; ^5^ Marine Eco‐Evo‐Devo Unit Okinawa Institute of Science and Technology Onna son Okinawa Japan

**Keywords:** adaptation, coral reef fish, gene duplication, opsins, spectral tuning, UV communication

## Abstract

Damselfishes (Pomacentridae) are one of the most behaviourally diverse, colourful and species‐rich reef fish families. One remarkable characteristic of damselfishes is their communication in ultraviolet (UV) light. Not only are they sensitive to UV, they are also prone to have UV‐reflective colours and patterns enabling social signalling. Using more than 50 species, we aimed to uncover the evolutionary history of UV colour and UV vision in damselfishes. All damselfishes had UV‐transmitting lenses, expressed the UV‐sensitive *SWS1* opsin gene, and most displayed UV‐reflective patterns and colours. We find evidence for several tuning events across the radiation, and while *SWS1* gene duplications are generally very rare among teleosts, our phylogenetic reconstructions uncovered two independent duplication events: one close to the base of the most species‐rich clade in the subfamily Pomacentrinae, and one in a single Chromis species. Using amino acid comparisons, we found that known spectral tuning sites were altered several times in parallel across the damselfish radiation (through sequence change and duplication followed by sequence change), causing repeated shifts in peak spectral absorbance of around 10 nm. Pomacentrinae damselfishes expressed either one or both copies of *SWS1*, likely to further finetune UV‐signal detection and differentiation. This highly advanced and modified UV vision among damselfishes, in particular the duplication of *SWS1* among Pomacentrinae, might be seen as a key evolutionary innovation that facilitated the evolution of the exuberant variety of UV‐reflectance traits and the diversification of this coral reef fish lineage.

## Graphical abstract

Duplication and tuning of the UV‐sensitive *SWS1* opsin in damselfishes as key evolutionary innovation.

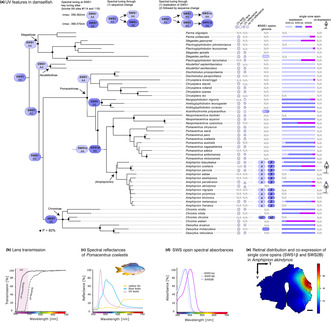

## 1 INTRODUCTION

Ultraviolet (UV) light (shorter than 400 nm) is visible to many animals that use UV vision for a number of behaviours such as orientation and navigation, foraging, prey and predator detection, or for intraspecific signalling and communication (reviewed in Cronin & Bok, 2016). This is also the case for the damselfishes (Pomacentridae), one of the most abundant, colourful and diverse reef fish families (Allen, 1991). Most damselfishes inhabit shallow, well‐lit and clear coral reefs where UV light is plentiful (Siebeck et al., 2006). Damselfishes have UV‐transmitting lenses (Siebeck & Marshall, 2007), and are sensitive to UV [for a review of microspectrophotometry (MSP) records of visual pigments, see (Marshall et al., 2006)]. Their UV sensitivity is thought to serve UV communication as shown for the Ambon damsel (*Pomacentrus amboinensis*) that uses facial UV patterns for species recognition (Siebeck et al., 2010). Indeed, many damselfishes have UV‐reflective body parts (Marshall, 2000b; Stieb et al., 2017). Also, as most predatory reef fish lack the UV‐sensitive visual pigment, these markings may serve as a ‘secret predator‐safe’ communication channel (Marshall & Cheney, 2011), such that their diversification may be released from constraint. Finally, the importance of UV vision in damselfishes is further supported by molecular studies: *SWS1* is expressed in all investigated species and is spectrally tuned by sequence evolution at key tuning sites, gene duplication and opsin co‐expression (simultaneous expression of different opsins within the same photoreceptor) (Hofmann et al., 2012; Luehrmann et al., 2018; Mitchell et al., 2021; Stieb et al., 2016, 2017, 2019, 2023).

Together with a vitamin A‐derived chromophore, visual opsins form the functional unit of the photoreceptor cell, the visual pigment mediating vision (Wald, 1968). Most vertebrates possess one rod opsin, the rhodopsin (RH1), often used for scotopic vision, and four basic types of cone opsins mediating colour vision: the short‐wavelength‐sensitive opsins with SWS1 absorbing in the UV (λ_max_ 347–383 nm) and SWS2 absorbing in the violet‐blue regions of the spectrum (λ_max_ 397–482 nm); the middle‐wavelength‐sensitive opsin rhodopsin 2 (RH2: λ_max_ 452–537 nm); and the long‐wavelength‐sensitive opsin (LWS: λ_max_ 501–573 nm) (Yokoyama, 2008). The evolutionary history of opsins in teleost fishes is especially diverse including many lineage‐specific and some species‐specific gene duplications and deletions (Musilova et al., 2021). The differential expression and co‐expression of these opsins (Hofmann et al., 2009), together with sequence evolution (Sugawara et al., 2005) and a switch in chromophore type (Munz & McFarland, 1977), facilitate spectral tuning to different photic environments and/or different visual tasks in fish (Bowmaker & Hunt, 2008; Carleton et al., 2020).

In damselfishes, opsin gene expression analyses in 40 species from four clades (Abudefdufinae, Chrominae, Pomacentrinae and Stegastinae) showed that all species expressed the *SWS1* gene (Luehrmann et al., 2018; Mitchell et al., 2021; Stieb et al., 2016, 2017, 2019, 2023). The *SWS1* (and *SWS2B*) opsin gene in damselfish has been shown to be expressed in single cones (Stieb et al., 2019). Further, across species, the largest functional variation at known spectral tuning sites was observed in SWS1 (Hofmann et al., 2012; Stieb et al., 2017). Moreover, a genomic study revealed that one Stegastinae species had only one, but one Chrominae species had two copies of the *SWS1* opsin (Musilova et al., 2019), an otherwise relatively rare duplication among teleost fishes (Lin et al., 2017; Musilova et al., 2019; Rennison et al., 2012). Interestingly, another *SWS1* duplication was discovered recently in anemonefish that was suggested to have occurred at the stem of the anemonefish radiation [tribe Amphiprionini (Tang et al., 2021)] with 11 anemonefish species having two copies present in their genome while expression data in one species (*Amphiprion ocellaris*) highlighted that only one copy was expressed (Mitchell et al., 2021). Only recently, transcriptomic data discovered two *SWS1* copies in *Pomacentrus australis* and *Amphiprion percula* (both belonging to Pomacentrinae) (Stieb et al., 2023). Thus, this is the first study (1) indicating that the duplication might have happened deeper in the Pomacentrinae evolution, and (2) showing a simultaneous expression of two *SWS1* duplicates in teleost fishes. Opsin gene duplications followed by sequence divergence are mostly associated with changes at key tuning sites among paralogues, and may thus lead to shifts in the peak absorbance (λ_max_) of the two copies of the visual pigment and, consequently, sensitivities to different wavelengths of light (Rennison et al., 2012; Yokoyama, 2008; Yokoyama et al., 2016). With gene duplications providing the potential for protein‐level innovations (reviewed in Taylor & Raes, 2005), opsin gene duplications and divergence can be subject to environmental heterogeneity as proposed by Rennison et al. (2012) or other ecological factors.

In this study, we aimed to present and understand the patterns of UV‐sensitive pigment evolution through tuning and duplication, and its role in UV communication and in the radiation of the species‐rich damselfishes. First, we hypothesised that sensitivity to UV and UV reflection – the factors mediating UV communication – is widespread or even omnipresent among the damselfish radiation. Combining published and new data allowed us to illustrate the presence of UV‐transmitting lenses, expression of the UV‐sensitive *SWS1* opsin gene (and *SWS1* duplications) and UV‐reflective colours and colour patterns among species spanning the damselfish phylogeny.

We further assumed that *SWS1* duplicates in anemonefish might show ontogenetic shifts in expression. This assumption comes from the observation that the duplication of *SWS1* was present in the genomes of 11 anemonefish species; however, expression data on two species showed that one species expressed only one but the other both duplicates (Mitchell et al., 2021; Stieb et al., 2019). Anemonefish form family groups and are sequential hermaphrodites with a size‐dependent hierarchy consisting of several smaller sexually immature individuals and a dominant sexually mature pair of which the largest individual is the female (Allen, 1975). Therefore, opsin expression in a developmental series spanning those life stages was measured in five anemonefish species combining new and published data (Stieb et al., 2023).

Next, we were interested in the evolutionary history of the *SWS1* duplication and whether it might qualify as a key innovation leading to increased diversification rates. Performing ancestral state reconstructions within damselfish species with knowledge of the SWS1 structure (genomic and/or transcriptomic data), we resolved duplication events of *SWS1* on a subset of the damselfish phylogeny. We then estimated diversification rates across the entire damselfish phylogeny to visualise whether evolutionary rate shifts coincided with *SWS1* duplication events.

Finally, we anticipated that *SWS1* duplicates may facilitate a functional mechanism for spectral tuning in the UV range. For this, we used estimates of the SWS1 visual pigments' spectral absorbance based on sequence structure.

## 2 METHODS

### 2.1 Specimen collection

All specimens were either collected from reefs surrounding North Stradbroke Island (Southern Queensland, Australia) or surrounding Lizard Island, Australia, using SCUBA and hand nets under the Great Barrier Reef Marine Park Permit (G12/35005.1) and the Queensland General Fisheries Permit (140763). Fish used were anaesthetised with an overdose of clove oil and killed by decapitation. All experimental procedures were approved by the University of Queensland Animal Ethics Committee (QBI/223/10/ARC/US AIRFORCE (NF) and QBI/192/13/ARC).

### 2.2 Lens transmission and spectral reflectance

The lens has been shown to be the primary light filter of the damselfish eye (Siebeck & Marshall, 2007). Here, we combined the lens transmittance data [the standard means of characterising ocular media transmission is to determine the wavelength at which 50% of the maximal transmittance (T50) was reached (Douglas & McGuigan, 1989)] from the literature [*n* = 38; (Siebeck & Marshall, 2007; Stieb et al., 2017, 2023)] to assess whether damselfish eyes are UV blocking (T50 > 400 nm) or UV transmitting (T50 < 400 nm). To measure the lens transmission, light from a pulsed xenon light source (Ocean Optics, PX2, USA) was directed through the lens mounted above a pinhole and into a quartz fibre‐optic cable coupled to a spectrometer (USB2000; Ocean Optics, Dunedin, USA), and 5–10 measurements were made per individual.

To determine whether damselfishes have UV colours and patterns, we measured anew the UV‐spectral reflectance for *A. ocellaris* [as per Marshall et al. (2003)] and compiled previous measurements for 27 species from the literature (Cheney & Marshall, 2009; Cortesi & Cheney, 2010; Marshall, 2000b; Siebeck, 2002; Stieb et al., 2017, 2023). UV colouration was defined as having a spectral reflection below 400 nm, following the colour categorisation in Marshall (2000b).

The spectral reflectance of different areas of the fish was measured at a 45° angle using a 200 nm bifurcated UV/visible optic fibre connected to a PX‐2 pulse xenon light source (Ocean Optics) and an Ocean Optics (Dunedin, FL, USA) USB2000 spectrophotometer attached to a laptop computer running OOIBASE32 (Ocean Optics). A Spectralon with 99% white reflectance standard was used to calibrate the percentage of light reflected at each wavelength from 300 to 800 nm. Spectral reflectance was obtained for five individuals (two males, three females) of *A. ocellaris* by measuring white, orange and black colour patches on the head and body taken from dorsal to ventral. Three measurements per area per individual were taken. As no differences within one colour were found within and between individuals, measurements per colour (white, orange and black) were subsequently averaged.

### 2.3 SWS1 opsin gene duplication and expression

To investigate the prevalence of *SWS1* duplications and the pattern of gene expression, we combined published opsin gene sequences (Sanger and whole genome sequencing) with available data on opsin gene expression from both quantitative real‐time polymerase chain reaction (qRT–PCR) and bulk retinal RNA sequencing (Hofmann et al., 2012; Luehrmann et al., 2018; Mitchell et al., 2021; Musilova et al., 2019; Stieb et al., 2016, 2017, 2019, 2023). The combined dataset comprised 52 damselfish species for sequence analysis and 40 species for gene expression analysis (Table S1). It is to note that within this dataset, we re‐screened our previously used transcriptomic data for damselfishes in general and anemonefishes in detail as *SWS1* opsin duplications had been identified in 11 anemonefish species in a genomic analysis (Mitchell et al., 2021) and expression of two *SWS1* copies in *Amphiprion percula*, *A. ocellaris* and *Pomacentrus australis* (Stieb et al., 2023). To further investigate the expression pattern of *SWS1* copies for anemonefishes in more detail, we produced retinal transcriptomes for different developmental stages (females, males and juveniles) for five anemonefish species as done for *Amphiprion akindynos* in our previous study (Stieb et al., 2019). For this, we completed a developmental series by combining published [*A. biaculeatus* (*n* = 2), *A. melanopus* (*n* = 2), *A. percula* (*n* = 2) and *A. perideraion* (*n* = 2) (Stieb et al., 2023)] with new retinal transcriptomes [*A. biaculeatus* (*n* = 4), *A. melanopus* (*n* = 8), *A. percula* (*n* = 2) and *A. perideraion* (*n* = 2)].

As Stieb et al. (2019) demonstrated damselfish single cones only express *SWS* (*SWS1* and *SWS2B*) opsin genes, whereas double cones (two single cones fused together) express *RH2* (*RH2A* and *RH2B*) and *LWS* opsin genes, we present the *SWS* opsin expression as a proportional fraction of total single‐cone expression.

In addition to the retinal transcriptomes of extra specimens of anemonefish, we used opsin sequence data for one specimen of *Plectroglyphidodon johnstonianus*. Due to low coverage, reads of the latter specimen could not be used to resolve opsin gene expression and were thus excluded from a previous study (Stieb et al., 2023).

#### 2.3.1 Transcriptomic sequencing and processing

Retinas were homogenised using a TissueLyser LT (Qiagen, Netherlands) and total RNA was extracted with the RNeasy Mini Kit (Qiagen, Netherlands), including an optional DNAse digestion step. RNA was quality‐checked with an Agilent 2100 BioAnalyzer 6000 NanoChip (Agilent Technologies, USA). RNAseq libraries were made using the TruSeq RNA Sample Preparation Kit v.2 (Illumina, San Diego, USA), and transcriptomes were sequenced as 125 bp paired reads on the Illumina platform (HiSeq2000 v4). Samples were multiplexed at 12 samples per lane, obtaining between 4 and 51 million sequenced reads per sample.

Transcriptomes were processed following previously published methods (Cortesi et al., 2015; de Busserolles et al., 2017) using the online Bioinformatics platform Galaxy v.1.0.4 (Research Computing Centre, The University of Queensland, Australia) (Afgan et al., 2015). In short, data were converted using FASTQ Groomer, quality checked using FastQC and trimmed using customised settings in Trimmomatic. Trinity was used for *de‐novo* assembly of transcripts, with a group pair distance of 250 bp, and minimum inchworm kmer coverage of 2. Further bioinformatics analyses were performed using Geneious software (Version 9.0.4). Assembled transcripts were then mapped to the known *SWS1* duplicates of reference species (Mitchell et al., 2021).

#### 2.3.2 Opsin gene expression

To analyse the differences in cone opsin gene expression, we mapped the unassembled filtered PE reads against the CDSs of genes extracted from the transcriptomes [as per Cortesi et al. (2015) and de Busserolles et al. (2017)]. Proportional gene expression was then calculated according to *T*_i_/*T*_all_ = *N*_i_/∑*N*_i_, where *T*_i_/*T*_all_ is the gene expression ratio for a given gene *T*_i_ normalised by the total genes expressed in all single cones or in all double cones *T*_all_, and *N*_i_ is the number of mapped reads for a given gene divided by its length.

### 2.4 SWS1 λ_max_ value predictions

For 52 damselfish species, variability in amino acid identity was examined at known SWS1 tuning sites [numbers referring to bovine rhodopsin (PDB accession no. 1U19)] located in the retinal binding pocket or within transmembrane regions I (sites 46, 49, 52), II (sites 86, 90, 91, 93, 97), III (sites 109, 113, 114, 116, 118) and VI (site 265) (Shi & Yokoyama, 2003; Yokoyama, 2008). For those λ_max_ estimates, we assumed that an A1 chromophore would be used as generally seen in marine fishes (Toyama et al., 2008). Damselfish SWS1 λ_max_ were estimated by comparing their amino acids to tuning sites of SWS1 opsins from fish where the spectral absorbance was gained from in‐vitro opsin protein expression studies or measured using MSP (for reference species, see Table S2). Individual site effects were then judged based on their polarity (polar or non‐polar), and estimated λ_max_ contributions were then added or subtracted from the known λ_max_ value of the closest matching species.

Because individual SWS1 site differences are usually not additive (Shi & Yokoyama, 2003), we could only make λ_max_ predictions for sequences with a high degree of tuning‐site homology with reference sequences and only when reasonable estimations of single‐site effects could be made based on clear differences found in reference sequences. However, this was rarely an issue, as almost all examined sequences matched with at least one model sequence. In cases with sequences matching more reference species resulting in different λ_max_ predictions, we used, if applicable, other damselfish reference species. However, in most cases, using different reference species did not produce λ_max_ shifts more than a few nanometres, and in all cases, did not alter whether a species has the short‐ or long‐shifted λ_max_ prediction for SWS1.

### 2.5 Phylogenetic reconstruction of the SWS1 duplication

To resolve the phylogenetic relationship of SWS1 opsins among the damselfish radiation, we first reconstructed maximum‐likelihood amino acid trees using PHYML (100 bootstrap iterations in MEGA 11) only for species (*n* = 30) for which the sequences originated from whole genome or transcriptome studies (Table S1). *SWS1* sequences gained from Sanger sequencing without cloning might represent hybrids between gene copies and were thus excluded from phylogenetic analyses. As outgroups, we used SWS1 sequences of the Japanese rice fish, *Oryzias latipes* (BAE78652.1), the Zebra mbuna, *Maylandia zebra* (NP_001297003.1), the Nile tilapia, *Oreochromis niloticus* (*ADW80527.1*), the Atlantic salmon, *Salmo salar* (AAP58324.1), the Zebrafish, *Danio rerio* (AAD24756.1), the Pouched lamprey, *Geotria australis* (AAR14684.1), the Green anole, *Anolis carolinensis* (AAD32621.1), and human, *Homo sapiens* (NM_001708.2). Next, we computed ancestral states (also in MEGA 11) for AA sites with special interest in those sites that were estimated to cause shifts in spectral absorbance (bovine # 114 and 118). Ancestral state reconstructions were performed with the full AA SWS1 sequence for reference species and with #114 and #118 removed for reference species to control for any bias introduced by those sites in the damselfish history.

Next, to visualise the most likely scenario of SWS1 evolution across the damselfish phylogeny, we reconstructed a maximum‐likelihood tree for 52 damselfish species with knowledge of SWS1 sequence structure. For 50 species, we downloaded a sequence matrix construction from *The Fish Tree of Life* (Rabosky et al., 2018) that is composed of 24 genes obtained using the gene‐baited approach of PyPHLAWD (Smith & Walker, 2019). Two species, *Pomacentrus wardi* and *Parma oligolepis*, were not covered in the dataset of *The Fish Tree of Life*. Therefore, we included the *rag 1* nuclear marker for *Pomacentrus wardi* [MW631536.1]. As no genetic markers were available for *Parma unifasciata*, we used markers available for *Parma oligolepis* as a surrogate to place *Parma unifasciata* in the phylogeny.

### 2.6 Diversification rate analyses

To estimate temporal and clade variation in the speciation rate within Pomacentridae, we used the Bayesian Analysis of Macroevolutionary Mixtures (BAMM 2.5) approach (Rabosky, 2014). BAMM uses a reversible‐jump Markov Chain Monte Carlo (MCMC) method to detect parts of the tree that share common evolutionary parameters of diversification and to identify core shifts in the rate of diversification (Rabosky et al., 2014). Our primary goal was to test if any speciation rate shift co‐occurred with the duplication of the *SWS1* gene. For that, we used the damselfish calibrated phylogeny from McCord et al. (2021) inferred based on 12 nuclear and mitochondrial gene sequences from 345 of the 423 damselfishes. To account for incomplete taxon sampling, we set BAMM analysis to assume for each genus a fraction of missing species according to Table S3. Appropriate priors were determined using the set BAMMpriors function of the BAMMtools 4.1.2 package (Rabosky et al., 2014) in the R environment (R Core Team, 2021). Four independent metropolis‐coupled MCMC chains of 1 × 10^9^ generations were run, sampling the parameters every 25,000 steps. By plotting the log‐likelihood values, we confirmed stationarity and determined the burn‐in. We also confirmed that the effective sample size (ESS) of both log‐likelihood and number of shift events was above 200.

## 3 RESULTS

### 3.1 Lens transmission and spectral reflectance

**FIGURE 1:**
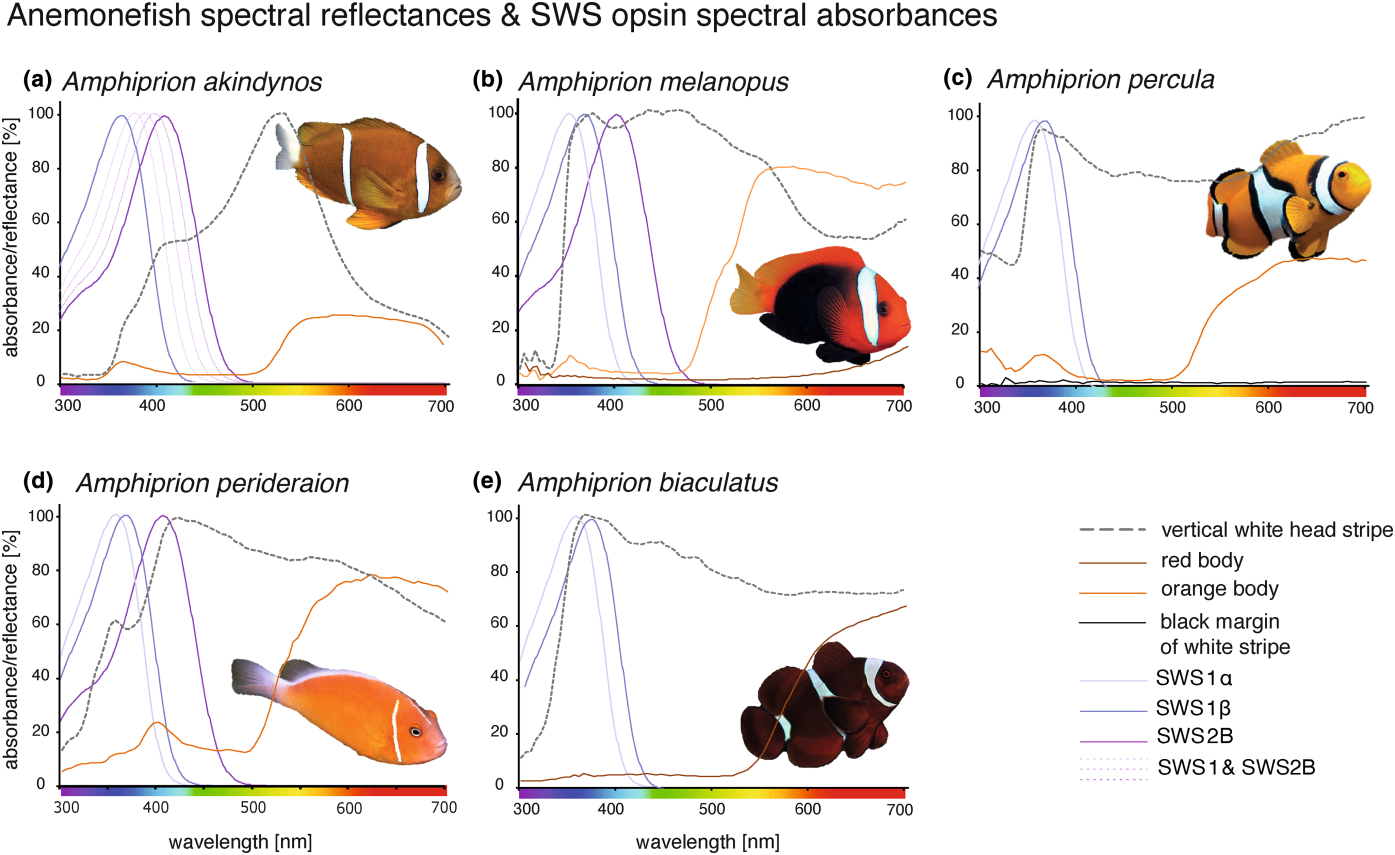
Five anemonefish species living in sympatry on the same reefs around Lizard Island, Northern Great Barrier Reef, with reflectance spectra of colours and spectral absorbance curves from expressed short‐wavelength‐sensitive visual pigments [SWS1α λ_max_: 356–360 nm and SWS1β λ_max_: 367–370 nm (estimates for SWS1 from this study); SWS2B λ_max_: 407 nm (Stieb et al., 2016)]. In the Barrier Reef anemonefish (*Amphiprion akindynos*), it is known that the ‘UV’ *SWS1* and ‘violet’ *SWS2B* opsins are co‐expressed within the same single cones resulting in intermediate absorbances (visualised by the dashed lines). References of spectral reflectance: (a) Stieb et al. (2019), (b–d) Stieb et al. (2023), (e) Marshall (2000b).

All 38 inspected damselfish species had UV‐transmitting lenses (Table S4). Twenty‐six out of 28 examined damselfish species had UV‐reflective body parts or patterns (Table 1). Reflectance measurements from five sympatric anemonefish species listed in Table 1, that can be found within only a few metres on the reefs surrounding Lizard Island, are shown in Figure 1. It is to be noted that white stripes have a black margin for all (but *A. perideraion*) anemonefish species listed here. Whilst the black margin is listed for those species in Table 1, it is only shown for *A. percula* in Figure 1. This is because the black stripe of most anemoenfish species is too narrow and measurements tend to either contain orange or white reflectance measurements from the neighbouring body colours.

**TABLE 1:** Spectral reflectance measurements of various body parts, including species‐specific patterns on the head or fins for 27 damselfish species.

	Colour categories																
	UV	UV/blue	UV‐hump/blue	Blue	Blue/green	Yellow	Orange	Red	Brown	White (UV)	Black	UV/green	UV/yellow	UV/orange	UV/blue/orange	UV/blue/green	UV reflection
**Abudefdufinae**																	
*Abudefduf sexfasciatus* ^e^																	
**Chrominae**																	
*Chromis viridis* ^a^																	
*Dascyllus aruanus* ^a^																	
*Dascyllus reticulatus* ^c^																	
**Pomacentrinae**																	
*Acanthochromis polyacanthus* ^c^																	
*Amblyglyphidodon curacao* ^a^ ^,^ ^c^																	
*Amblyglyphidodon leucogaster* ^a^ ^,^ ^c^																	
*Chrysiptera cyanea* ^a^ ^,^ ^c^																	
*Chrysiptera rollandi* ^c^																	
*Dischistodus prosopotaenia* ^a^ ^,^ ^c^																	
*Neopomacentrus azysron* ^a^ ^,^ ^c^																	
*Neoglyphidodon nigroris* ^c^																	
*Pomacentrus amboinensis* ^b^																	
*Pomacentrus chrysurus* ^a^																	
*Pomacentrus coelestis* ^c^																	
*Pomacentrus moluccensis* ^a^																	
*Pomacentrus nagasakiensis* ^c^																	
*Pomacentrus pavo* ^c^																	
*Pomacentrus wardi* ^a^																	
**(Amphiprionini)**																	
*Amphiprion akindynos* ^d^											Black margin			Orange body			
*Amphiprion biaculeatus* ^a^							Orange body			White stripe	White stripes						
*Amphiprion melanopus* ^e^								Red body		White stripes	Black margin			Orange body			
*Amphiprion ocellaris* ^f^																	
*Amphiprion percula* ^e^										White stripes	Black margin			Orange body			
*Amphiprion perideraion* ^e^			White stripes							White stripes				Orange body			
**Stegastinae**																	
*Plectroglyphidodon lacrymatus* ^e^																	
*Stegastes apicalis* ^a^																	
*Stegastes partitus* ^a^																	

*Note*: Reflectance data are summarised in colour categories as defined by (Marshall, 2000b). Highlighted in the last column are species reflecting in the UV (<400 nm). Only two species had no UV reflection. For five of the six listed anemonefish species (selection limited to species that occur in sympatry at reefs surrounding Lizard Island), reflectance data for the colour categories are shown in Figure 1, and regions where measurements have been taken are indicated in the grey‐shaded area.

^a^ Marshall (2000b).

^b^ Siebeck et al. (2010).

^c^ Stieb et al. (2017).

^d^ Stieb et al. (2019).

^e^ Stieb et al. (2023).

^f^ This study.

### 3.2 SWS1 opsin gene duplication and expression

**FIGURE 2:**
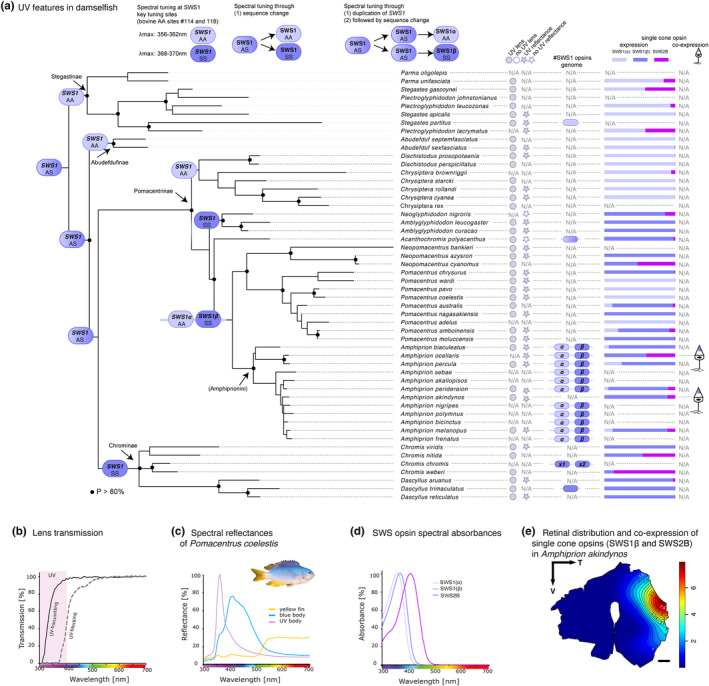
UV features underpinning the UV communication system in damselfishes (Pomacentridae). All damselfish species investigated (*n* = 51) had UV‐transmitting lenses (*n* = 40), expressed *SWS1* (*n* = 40) and some also *SWS2B*, and most species reflected in the UV (*n* = 26 out of 28). The variable single‐cone (co)expression profile enables the fine‐tuning of the UV‐sensitive photoreceptors, which might serve UV‐based communication. (a) Damselfish phylogeny [maximum‐likelihood tree modified from *The Fish Tree of Life* (Rabosky et al., 2018); in addition to the *rag 1* nuclear marker for *Pomacentrus wardi (MW631536.1*; as no genetic markers were available for *Parma unifasciata*, we used markers for *Parma oligolepis* as a surrogate to place *Parma unifasciata* in the phylogeny) showing the most likely evolution of SWS1, presence of UV‐reflective body parts (UV‐reflectance, see also Table 1) (Cheney & Marshall, 2009; Cortesi & Cheney, 2010; Marshall, 2000a; Siebeck, 2002; Stieb et al., 2017, 2023), UV‐transmissive lenses (UV lenses, see also Table S4) (Siebeck & Marshall, 2007; Stieb et al., 2017, 2023), and the presence of *SWS1* opsin genes in the genome (see Table S1 for Genbank accession #). It also shows the proportional single cone opsin expression (for a summary expression, see Table S4) with the violet‐sensitive *SWS2B* and the UV‐sensitive *SWS1* opsins (the ‘short’ and the ‘long’), and single cone co‐expression (Mitchell et al., 2021; Stieb et al., 2019). (b) Lens transmission curves show an example of a damselfish (*Dascyllus aruanus*) UV‐transmitting lens (continuous black line) vs. a UV‐blocking lens from a predatory reef fish (*Plectropomus leopardus*) (dashed grey line). (c) Reflectance spectra of an exemplary damselfish species, *Pomacentrus coelestis*, reflecting in UV. (b) and (c) are adapted from (Stieb et al., 2017). (d) Spectral absorbance curves from short‐wavelength‐sensitive visual pigments [SWS1α λ_max_: 356–360 nm and SWS1β λ_max_: 367–370 nm (estimates for SWS1 from this study); SWS2B λ_max_: 407 nm (Stieb et al., 2016)]. (e) Single‐cone opsin expression revealed by fluorescent in situ hybridization (FISH) in *Amphiprion akindynos* [adapted from Stieb et al. (2019)]. Topographic distribution of FISH‐based opsin gene expression shows co‐expression of *SWS1β* and *SWS2B*, forming a small dorso‐temporal area. The black lines represent iso‐density contours, and values are expressed in densities × 10^3^ cells/mm^2^. The black arrow indicates the orientation of the retina. T = temporal, V = ventral. Scale bar: 1 mm.

For the 15 species with whole genome data, *Stegastes partitus* (Stegastinae), *Dascyllus trimaculatus* (Chrominae) and *Acanthochromis polyacanthus* (Pomacentrinae) were found to have one *SWS1* gene, whilst *Chromis chromis* (Chrominae) and the 11 anemonefish species (tribe Amphiprionini: *Amphiprion akallopisos*, *A. biaculeatus*, *A. bicinctus*, *A. frenatus*, *A. melanopus*, *A. nigripes*, *A. ocellaris*, *A. percula*, *A. perideraion*, *A. polymnus* and *A. sebae*) were found to have two copies of the gene (Figure 2).

For the 40 damselfish species with expression data, all species expressed at least one *SWS1* gene, with six species expressing two copies (*Pomacentrus amboinensis*, *P. australis*, *Amphiprion biaculeatus*, *A. melanopus*, *A. percula* and *A. perideraion*) (Figure 2; Table S4).

For five anemonefish species (*A. ocellaris*, *A. biaculeatus*, *A. melanopus*, *A. percula*, and *A. perideraion*), genomic and expression data were available, uncovering that two copies were present in the genome [also see Mitchell et al., (2021)] and all but *A. ocellaris* expressed both copies. Further, for the anemonefish, no expression differences were identified between the developmental stages (Table S4).

### 3.3 SWS1 λ_max_ predictions, phylogenetic reconstruction of SWS1

The 52 damselfish species had a combined total of 67 SWS1 opsin sequences. Based on their amino acid sequences, we were able to estimate λ_max_ for all those opsins, except for the SWS1 opsins found in *Dascyllus aruanus*, *Pomacentrus adelus* and *Chromis chromis* as these contained ambiguous positions (Table S2). Changes at two known SWS1 tuning sites (Shi & Yokoyama, 2003; Yokoyama, 2008) (Table S2), A114S and A118S (with the latter known to induce a +5 nm shift in opsin λ_max_), were observed across the damselfish species. Those were responsible for the major estimated shift of ~10 nm in λ_max_ (based on comparison to reference species). Whilst the SWS1 with the shorter λ_max_ of 356–362 nm had A114 and A118, the SWS1 with the longer λ_max_ of 368–370 nm had S114 and S118.

**FIGURE 3:**
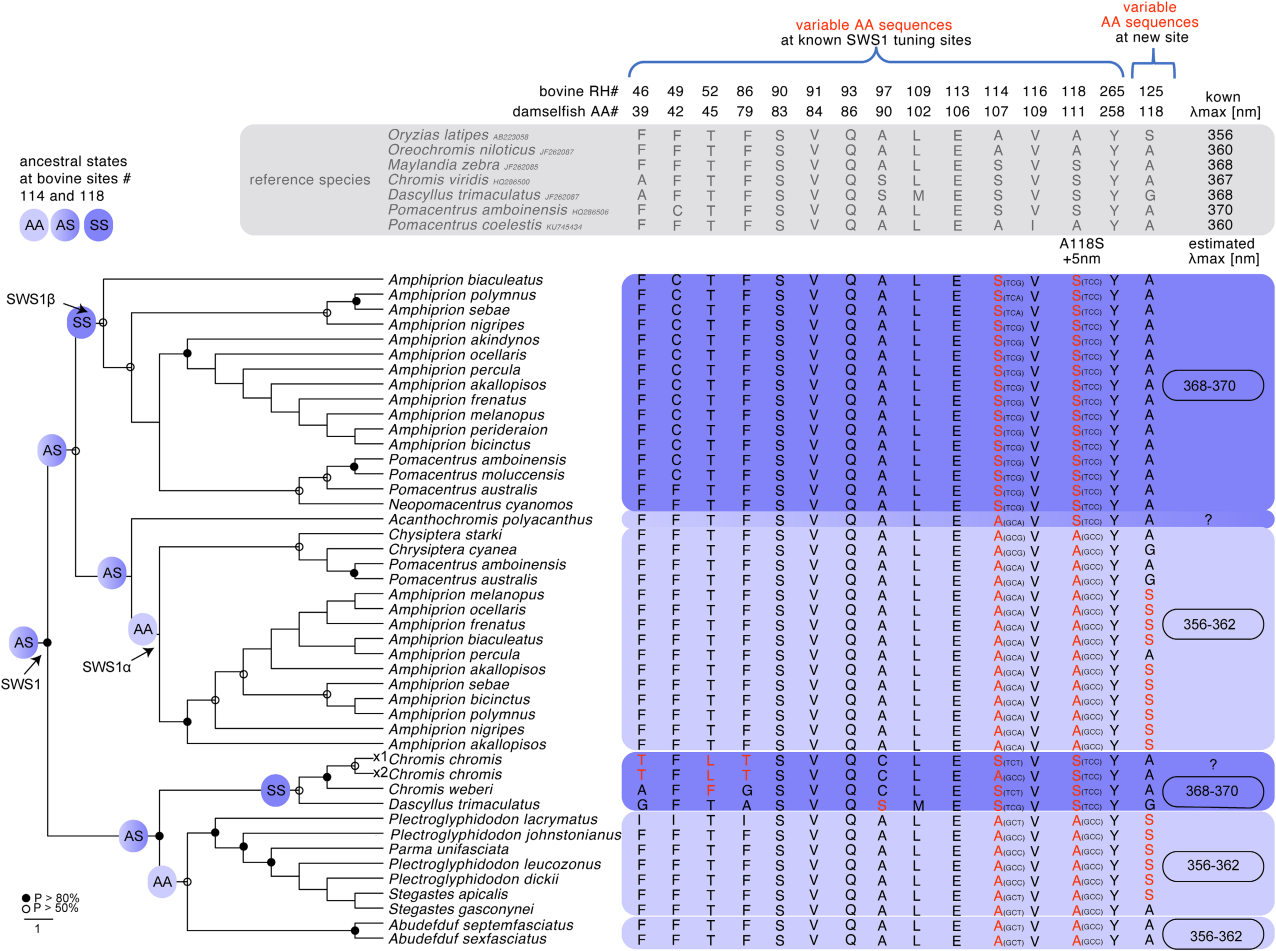
The damselfish (Pomacentridae) maximum likelihood phylogenetic tree of the SWS1 opsin protein (AA) with amino acid translations of the relevant sites for spectral tuning, and ancestral states for AA sites #114 and #118. Only AA changes at transmembrane or retinal chromophore binding pocket regions that have previously been determined as tuning sites (Dungan et al., 2016; Hunt et al., 2001; Takahashi & Ebrey, 2003; Wilkie et al., 2000; Yokoyama, 2008; Yokoyama et al., 1999, 2007) are shown with changes in polarity (polar vs non‐polar) in red. One additional retinal binding pocket site (#125) with polarity changes that has been described as a potentially relevant site for visual tuning in Mitchell et al. (2021) is also displayed. Peak spectral absorbances (λ_max_) calculations are based on SWS1 sequence comparison to reference species with known pure protein spectral absorbance (λ_max_) [gained from in‐vitro opsin protein expression studies for *Oreochromis niloticus* (Parry et al., 2005), *Metriaclima zebra* (Spady et al., 2006), and *Oryzias latipes* (Matsumoto et al., 2014)], and to other damselfish species having a very similar sequence in known tuning sites and with known λ_max_ gained from microspectrophotometry [*Pomacentrus amboinensis* (Siebeck et al., 2010), *P. coelestis*, and *Dascyllus trimaculatus* (McFarland & Loew, 1994)]. Only damselfish species with opsin sequences obtained from genomic or transcriptomic studies are displayed (*n* = 30). For protein structure of relevant sites in all species, see Table S2. Ancestral reconstructions of key tuning sites #114 and #118 suggest that the damselfish ancestor, and basal nodes, most likely had an SWS1 (AS) intermediate between the ‘short’ (AA) and the ‘long’ (SS) SWS1 of today's damselfish species.

Phylogenetic reconstruction of the SWS1 protein tree (Figure 3) revealed that the damselfish ancestor, and several basal nodes had an intermediate SWS1 with A114 and S118. Parallel sequence changes of S118A towards the SWS1 with a shorter λ_max_ (356–362 nm) were observed in Stegastinae and Abudefdudinae. In contrast, a sequence change of A114S towards the SWS1 with a longer λ_max_ (368–370 nm) appeared in Chrominae. Also, within Chrominae, *Chromis chromis* had two SWS1 duplicates (SWS1x1 and SWS2x2), which are likely to be 3–9 nm apart based on A114S. Phylogenetic analyses further revealed that SWS1 duplicated within Pomacentrinae (with species possessing one or the other or both copies), followed by sequence changes leading to one paralogue (SWS1α) having again the short λ_max_ (356–362 nm) and one having the long λ_max_ (368–370 nm). It is to be noted that ancestral reconstructions at sites #114 and #118 did not change when outgroup species had information at those sites removed. Mapping the SWS1 evolution (based on species with genomic and/or transcriptomic information on *SWS1* structure) to the more comprehensive damselfish phylogeny from McCord et al. (2021), the main duplication event of SWS1 is likely to have occurred within the Pomacentrinae radiation at the split from Pomacentrinae 1 (Chrysiptera, Dischistodus, Pomachromis and Cheiliprion), 2 (*Hemiglyphidodon*, *Amblyglyphidon*, *Acanthochromis*, *Altrichthys* and *Neoglyphidodon*), from 3 (*Pristotis*, *Teixeirichthys* and *Neopomacentrus*), 4 (Amphiprionini) and 5 (*Pomacentrus and Amblypomacentrus*).

### 3.4 Diversification rate analyses

**FIGURE 4:**
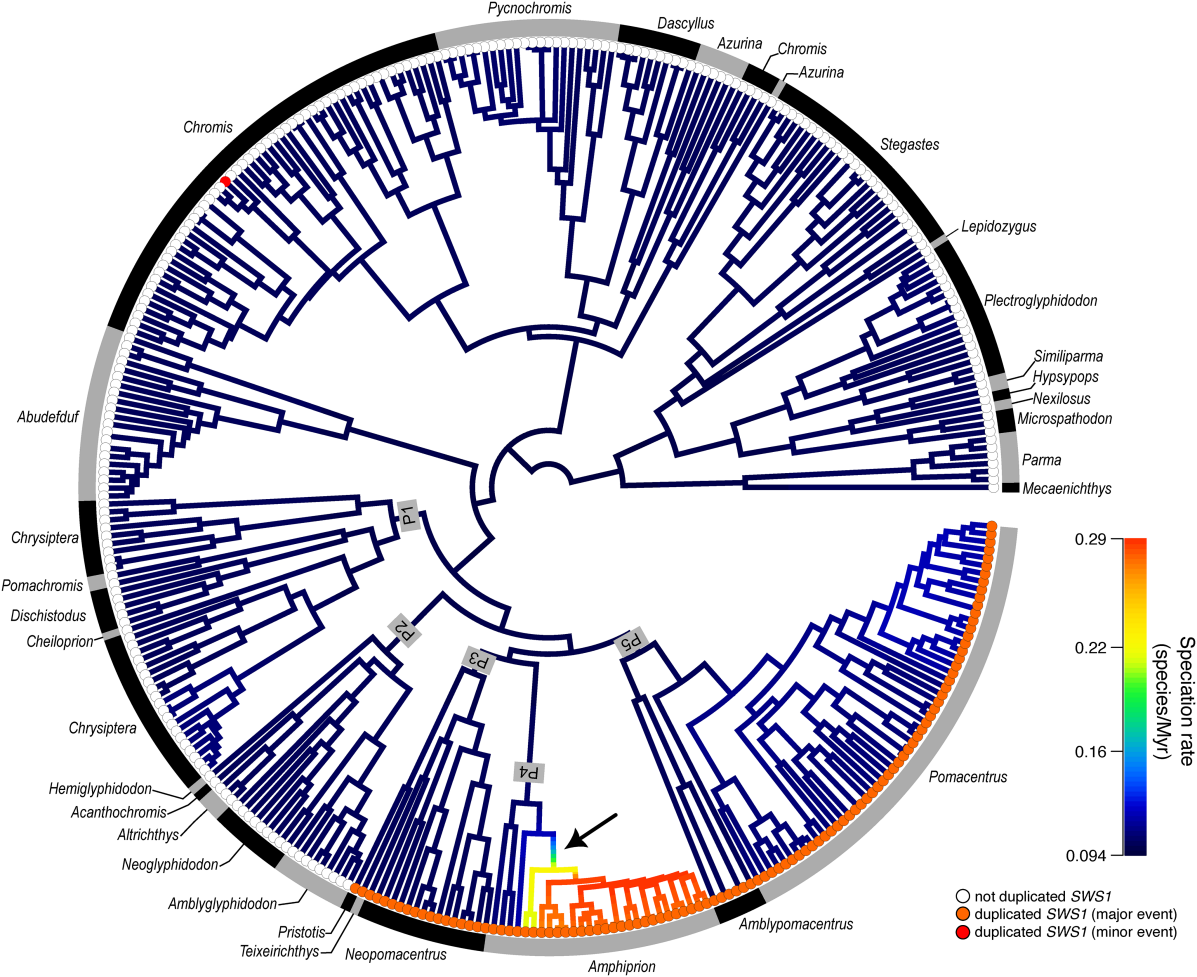
Diversification rates throughout the history of Pomacentridae using the phylogenetic tree of McCord et al. (2021) highlighting that elevated rates are only found within a subclade of Amphiprionini (see arrow). The main duplication event of *SWS1* (species having the main *SWS1* duplication are indicated with an orange dot at the tip) is likely to have occurred within the Pomacentrinae radiation at the split between the clade composed of Pomacentrinae 1 (P1: Chrysiptera, Dischistodus, Pomachromis and Cheiliprion), 2 (P2: *Hemiglyphidodon*, *Amblyglyphidon*, *Acanthochromis*, *Altrichthys* and *Neoglyphidodon*), and the clade composed of 3 (P3: *Pristotis*, *Teixeirichthys* and *Neopomacentrus*), 4 (P4: Amphiprionini) and 5 (P5: *Pomacentrus* and *Amblypomacentrus*). The minor duplication event has so far only been reported for *Chromis chromis* (indicated by a red dot at the tip). For the same tree with all tip labels (i.e., species names), see Figure S1.

Calculating average diversification rates across damselfish trees from BAMM reconstructions revealed that the speciation rate is overall homogenous (Figure 4 and Figure S1), except for a shift towards higher rates at the branch where an internal lineage of Amphiprionini emerged (13–8.2 Ma). Speciation rates remained constant in this clade until the present but higher than in the background. This clade belongs to a major lineage that experienced a duplication of *SWS1*.

## 4 DISCUSSION

### 4.1 UV vision in damselfishes

In this study, we investigated spectral tuning mechanisms and the evolutionary history of the UV‐sensitive *SWS1* opsin in damselfishes. All investigated species had UV‐transmitting lenses and express the UV‐sensitive *SWS1* gene (Figure 2 and Table S4). This UV‐sensitivity, together with most species reflecting in the UV (Table 1), underpins the role of UV communication in damselfishes. Most importantly, we uncovered two duplication events in SWS1 at various organisational levels (species, genus). Among the SWS1 evolution, we found that spectral tuning sites were altered in parallel over time, causing a shift in peak spectral absorbance of around 10 nm (Figure 3 and Table S2). This shift was either observed by sequence change only (Stegastinae, Abudefdufinae, Chrominae and *Chrysiptera* /*Dischistodus* species) or by duplication followed by sequence change (Pomacentrinae including Amphiprionini) (Figure 2). Particularly noteworthy is the fact that within Pomacentrinae, many species expressed both copies, adding another dimensionality to their UV vision. With *SWS1* being the least often duplicated opsin in teleosts, with a maximum of two but in most cases one copy present (Musilova et al., 2021), the evidence for two duplication events of *SWS1* in damselfishes supports the importance of UV vision to this group. In this context, it is to note that part of the previously described structural diversity of the *SWS1* opsin gene (Hofmann et al., 2012; Stieb et al., 2017) can be attributed to the newly discovered duplication (Figure 3 and Table S2).

### 4.2 SWS1: Parallel spectral tuning and duplication

In this study, we found several cases of parallel evolution at SWS1 spectral tuning sites and two independent *SWS1* duplication events in damselfishes. A species‐specific duplication was uncovered for *Chromis chromis* (no expression data available) with a likely shift in spectral sensitivities of >3 nm between copies (Table S2). Further, SWS1 in present‐day damselfish species is either short (λ_max_ ~ 360 nm) or long (λ_max_ ~ 370 nm) shifted with a difference of ~10 nm (Figures 2 and 3, Table S2). Importantly, these shifts in λ_max_ sometimes occurred through a change of the single ancestral gene copy, and sometimes after duplication through a change of one of the two new copies. It should be noted that λ_max_ estimates are calculated based on amino acid changes at known spectral tuning sites; however, we identified several additional amino acid changes in the retinal binding pocket or transmembrane regions that have not been described as spectral tuning sites yet, but that might effectively alter the λ_max_ of SWS1 (Table S2). Of these, site 125 within the retinal binding pocket region showed a shift in polarity. That was S125A, with Stegastinae having S125 and Chrominae having A125. The same site showed convergent changes in one of the SWS1 duplicates in each of *Chromis chromis* and anemonefish (also see Mitchell et al., 2021). Thus, AA site 125 might be a putative tuning site that we urge to be tested empirically e.g. by protein spectral absorbance (λ_max_) gained through in‐vitro opsin protein expression studies as a tuning site for future studies.

Ancestral reconstructions of SWS1 at the key tuning sites (#114 and #118) imply that the damselfish ancestor, as well as basal nodes, most likely had a SWS1 between the ‘short’ and the ‘long’ SWS1 of today's damselfish species (Figure 3). Merging the SWS1 evolution (AA tree) with the damselfish phylogeny, we suggest a scenario in which parallel evolution from a ‘medium’ (AS) towards a ‘short’ (AA) respectively ‘long’ SWS1 (SS) occurred through sequence changes as well as through duplication followed by sequence changes (Figure 2). Sequence changes towards the ‘short’ SWS1 occurred in Stegastinae, Abudefdufinae and *Chrysiptera* /*Dischistodus* species; a sequence change towards the ‘long’ SWS1 occurred in Chrominae. Further, the main duplication event of SWS1 is likely to have occurred within the Pomacentrinae radiation (see Figure 2, split *Neopomacentrus*, *Pomacentrus* and *Amphiprion* from the rest). This duplication resulted again in a ‘short’ and a ‘long’ SWS1 copy with species expressing one or the other or both paralogues. Notably, the duplication event in *Chromis chromis* might be more widespread among other Atlantic *Chromis* species. *Chromis chromis* (Mediterranean chromis) occurs in the Eastern Atlantic and Mediterranean, and several related species occur in the Eastern Atlantic; all other sampled *Chromis* species from this study were sampled from one reef in the Pacific (Northern Great Barrier Reef).

Most animals that are UV‐sensitive possess a single UV‐sensitive SWS visual pigment incorporated into a tri‐ or tetrachromatic colour vision system [for a review see Cronin & Bok (2016)]. However, terrestrial and marine arthropods are known to have multiple SWS opsins with distinct UV sensitivities that are simultaneously expressed (Bok et al., 2014; Henze & Oakley, 2015; Kashiyama et al., 2009; Marshall & Oberwinkler, 1999). While vertebrates typically only possess one copy of *SWS1*, several species of teleosts are reported to have up to two copies present in their genome (Cronin & Bok, 2016). In general, teleosts have an elevated number of duplicated opsins compared to other vertebrates, covering a broad spectral range from UV to red wavelengths (Hunt et al., 2014). However, most duplicates in teleosts are present in the short‐ to medium‐wavelength‐sensitive opsins (*SWS2* and *RH2*) tuned to the most frequent, blue‐green part of the aquatic light spectrum (Musilova et al., 2019). *SWS1* duplications are only found in very few fish species [1 out of 41 (Rennison et al., 2012), 1 out of 56 (Lin et al., 2017) and 12 out of 101 (Musilova et al., 2019) species investigated]. Even if a species has two *SWS1* copies within its genome, they might not be expressed simultaneously or at all. For example, the blackbar soldierfish (*Myripristis jacobus*) does not express either of its two copies (Musilova et al., 2019). Also, only one copy is expressed at the juvenile stage in Atlantic salmon (*Salmo salar*; Kunz et al., 1994) and at the adult stages in the Rainbow trout (*Oncorhynchus mykiss*; Cheng & Flamarique, 2007), the Ayu smelt (*Plecoglossus altivelis*; Minamoto & Shimizu, 2005) and the False‐clown anemonefish (*A. ocellaris*; Mitchell et al., 2021). Only one species so far, the Spotted gar (*Lepisosteus oculatus*), has been found to express both copies in its retina (Sukeena et al., 2016). Considering how rare *SWS1* duplications are and how seldom both copies are expressed simultaneously, it is remarkable to find several duplication events and the simultaneous expression of both genes in damselfishes. We hypothesisethat especially the duplication [dating back ~40 mya; for a time‐calibrated tree see McCord et al. (2021)] within the major, relatively recent and strictly coral‐reef associated radiation of Pomacentrinae (James Cooper et al., 2009) might qualify as a key innovation (Simpson, 1953) that opened up yet greater dimensionality in UV communication to the descendants of this lineage (based on diversity in UV sensitivity and UV colour traits). Indeed, two shifts in the rate of cladogenesis were identified within the Pomacentrinae (based on 187 sampled species), one at the base of the *Pomacentrus* (based on 27 sampled species) lineage and the other at the base of the *Amphiprionini* (based on 23 sampled species) (Cowman & Bellwood, 2011). In comparison, evolutionary rates estimated by BAMM based on the phylogenetic tree of McCord et al. (2021) (based on 345 sampled species) show an increase in the rate of speciation only in a subclade of Amphiprionini (based on 28 sampled species) but not in *Pomacentrus* (based on 59 sampled species) (Figure 4 and Figure S1). However, as our approach for dealing with missing data, i.e. missing species, was biased towards a higher number of missing species in species‐rich clades like Pomacentrinae (see Table S3), the detection of rate shifts in those clades could be affected. However, the present data suggests that the *SWS1* duplication alone is unlikely to have increased speciation in Amphiprionini. Interestingly, the lifestyle of anemonefishes, namely the mutualism with sea anemones (Litsios et al., 2012), and the dietary ecotype, mostly feeding in the water column on zooplankton, are linked to an elevated diversification rate in anemonefishes (McCord et al., 2021). In this context, it is interesting to note that visual models on an anemonefish visual system suggest that *SWS1* may serve to detect zooplankton, the host anemone and conspecifics (Stieb et al., 2019), with the latter supported by behavioural studies (Mitchell et al., 2022).

### 4.3 UV sensitivity: UV‐transmitting lenses and the overall expression of *SWS1*

Among the many fish that share the same UV‐rich coral reef environment, not all communicate in, or are sensitive to UV. Among the hundreds of reef fishes investigated so far, roughly half of the species, including all of the 56 damselfish species investigated (Siebeck & Marshall, 2007; Stieb et al., 2017, 2019, this study), had UV‐transmitting lenses (see Figure 2a for species used for this study and 2C for an example of a damselfish UV‐transmitting lens). However, UV‐transmitting lenses do not necessarily imply UV sensitivity. Indeed, measurements of visual pigment absorbance in over 60 reef fish species indicated that only very few species have a λ_max_ in the UV range [for a review of MSP records of visual pigments see Marshall et al. (2006)]. Again, damselfish are notable as most were found to have UV‐sensitive single cones. Those findings are supported by opsin gene expression data. Expression of *SWS1* is extremely rare in other reef fish families such as wrasses, surgeonfishes and cardinalfishes (Cortesi et al., 2020), all inhabiting the same shallow water reefs as the here investigated damselfish species. In comparison, the investigated 40 damselfish species belonging to four out of the five major damselfish lineages, all expressed the UV‐sensitive *SWS1* opsin (Figure 2a). Remarkably, seven species even expressed two copies, with six species (*P. amboinensis*, *P. australis*, *A. biaculeatus*, *A. melanopus*, *A. percula* and *A. perideraion*) expressing two copies with differentiated absorption spectra.

### 4.4 Biological significance of UV vision in damselfishes

UV sensitivity is known to increase the detectability of UV‐absorbing or scattering zooplankton (Browman et al., 1994; Loew et al., 1993; Novales Flamarique, 2016; Yoshimatsu et al., 2020). In freshwater cichlids, expression of *SWS1* was related to zooplanktivorous feeding (Hofmann et al., 2009, 2010). With many damselfish species being zooplanktivores (www.fishbase.org), *SWS1* expression may also enhance their foraging efficiency. This has been shown to be the case for larval and juvenile damselfishes (Job & Bellwood, 2007), and should be tested for adult stages in the future. However, among the damselfish opsin gene expression, only *LWS* was correlated to diet, with herbivorous species having an increased expression (Stieb et al., 2017).

UV cues are known to play major roles in animal signalling, including aggression, mate choice and species recognition (reviewed by Tovée, 1995). UV signalling in fishes is reported for guppies (Smith, 2002) and sticklebacks (Rick & Bakker, 2008a, 2008b). Damselfishes evidently have a UV‐communication system (Losey, 2003; Marshall & Cheney, 2011; Siebeck et al., 2006, 2010). Almost all (24 out of 26) species investigated so far have skin that reflects in the UV (for a summary, see Figure 2a and for more detail on colours and UV components, see Table 1; for an exemplary reflectance profile of damselfish with UV reflectance, see Figure 2b). One of the most astonishing examples of high‐level species differentiation based on UV signals comes from two species of almost identical‐looking yellow damselfishes (the Ambon damsel, *Pomacentrus amboinensis* and the Lemon damsel, *P. moluccensis*) that co‐occur in similar, often overlapping habitats. The Ambon damsel is able to distinguish between species based on subtle differences in UV‐reflective facial patterns (Siebeck et al., 2010). Moreover, they utilise that the complex facial patterns differ among individuals to differentiate between individual conspecifics. This suggests that fine‐scale UV patterns may not only be involved in species recognition but also provide important information about individuals and perhaps their social status. The striking UV reflectance in many species, together with the poor UV transmission in water, suggests that damselfishes may profit from a close‐range communication channel that might be invisible to ‘UV‐blind’ predators and concealed from other spectators at a distance (Losey, 2003; Marshall & Cheney, 2011; Siebeck et al., 2006). As many damselfish species co‐occur in the same habitat, and differences in fine‐scale UV patterns not only between different species but also among individuals of the same species may be more common, UV patterning may, in general, be used for differentiation between heterospecific and conspecific fish. The biological relevance for some damselfish species expressing two distinct UV‐sensitive opsins is speculative at this time but may help detect conspecifics based on fine‐scaled UV‐reflective patterns. For this purpose, it will be interesting to further study the intraretinal distribution and possible coexpression of *SWS1* copies. However, the high similarity between orthologues [>96% (Mitchell et al., 2021)] poses a challenge for commonly used techniques such as fluorescence in‐situ hybridisation, but might be overcome with single cell RNAseq of spatially localised target photoreceptors in the future.

Anemonefishes seem to be of particular interest regarding visual communication.Anemonefish typically have a striking appearance with UV‐white stripes and orange/red body colourations (Figure 1) (Cortesi et al., 2022). Among damselfishes, they are the group with the highest levels of long(red)‐wavelength‐sensitive (*LWS*) opsin gene expression, which is thought to be involved in social signalling based on their orange‐to‐red colouration (Stieb et al., 2023). They are also special regarding their UV‐sensitive visual pigments, as all anemonefish species analysed so far have two spectrally distinct SWS1 copies in their genomes (Mitchell et al., 2021). While no expression differences were identified between anemonefish developmental stages [a finding already reported before (Stieb et al., 2019)], inter‐specific expression differences were present. The six species analysed in this study, with five of them living in sympatry often within only a few metres from one another (Figure 1), showed highly variable single‐cone opsin expression profiles (Figure 2): species expressing one *SWS1* together with *SWS2B* (*A. akindynos* and *A. ocellaris*), species expressing both spectrally distinct *SWS1* copies (*A. biaculeatus* and *A. percula*), and species expressing both *SWS1* duplicates together with *SWS2B* (*A. melanopus* and *A. perideraion*). Their UV‐based orange‐white striped skin, together with a visual system tuned to enhance the contrast of their stripes, might be used for conspecific detection, to communicate their status to family members, or to convey that a host anemone has been occupied to members of rival groups from nearby anemones. Support for the former hypothesis comes from a recent behavioural study showing UV reflectance is an important indicator of dominance status in *A. akindynos* (Mitchell et al., 2022). Also, in *A. akindynos*, a localised area in the dorso‐temporal region (i.e., looking forward) of the retina was found to coexpress the ‘UV’ *SWS1* and ‘violet’ *SWS2B* opsins within the same single cones (Figure 2e) (Stieb et al., 2019). Using theoretical visual models from the perspective of the anemonefish, it suggested that this improves the detection of conspecifics by increasing the contrast of white stripes against the orange body colouration (Stieb et al., 2019). Anemonefishes that live in sympatry might indeed use their species‐specific variable set of single‐cone expression and fine differences in their UV pattern for conspecific detection among similarly looking anemonefish species (Figure 1).

## 5 CONCLUSION

The ubiquitous expression of the UV‐sensitive *SWS1* opsin gene, together with widespread UV colours and patterns found across the damselfish radiation, makes these coral reef fishes a group of special interest with regard to UV communication. The multiple parallel spectral tuning of SWS1, together with its duplication within the Pomacentrinae radiation, especially in Amphiprionini, an otherwise rare phenomenon across teleosts, might qualify as a key evolutionary innovation that led to the variety of UV‐colour traits and the diversification of this strictly coral reef‐associated fish radiation.

## AUTHOR CONTRIBUTIONS

**Sara M. Stieb:** Conceptualization (lead); data curation (lead); formal analysis (equal); investigation (equal); methodology (equal); validation (equal); visualization (lead); writing – original draft (lead); writing – review and editing (equal). **Fabio Cortesi:** Conceptualization (supporting); formal analysis (equal); investigation (equal); methodology (equal); validation (equal); writing – review and editing (supporting). **Laurie Mitchell:** Formal analysis (equal); investigation (equal); methodology (equal); validation (equal); writing – review and editing (supporting). **Luiz Jardim de Queiroz:** Formal analysis (equal); investigation (equal); methodology (equal); validation (equal); visualization (equal); writing – review and editing (supporting). **N. Justin Marshall:** Conceptualization (equal); funding acquisition (lead); supervision (supporting); validation (equal); writing – review and editing (supporting). **Ole Seehausen:** Conceptualization (equal); formal analysis (equal); methodology (equal); project administration (lead); supervision (lead); validation (equal); visualization (supporting); writing – review and editing (equal).

## CONFLICT OF INTEREST STATEMENT

There is no conflict of interest.

## Supporting information


Appendix S1.

Appendix S2.

## Data Availability

New opsin gene sequences have been deposited in the GenBank database (https://www.ncbi.nlm.nih.gov/genbank/), and accession numbers are listed in Table S1. All alignments used to reconstruct gene and protein trees are provided in the supplementary data. Raw reflectance measurements of *Amphiprion ocellaris* provided as Appendix S2.
