# Supplementary material for: Short‐wavelength‐sensitive 1 (SWS1) opsin gene duplications and parallel visual pigment tuning support ultraviolet communication in damselfishes (Pomacentridae)
Source: Ecol Evol. 2024 Apr 16;14(4):e11186. doi: 10.1002/ece3.11186 (PMC11019301; doi:10.1002/ece3.11186)
Supplement: Supplementary file 1 — Appendix S1. [file ECE3-14-e11186-s001.docx]

**Supplementary Figures and Tables**

**Table S1.** Summary of *SWS1* opsin genes and Genbank accession numbers (with numbers acquired in this study are bold) used in this study reaching a total of 52 damselfish species for sequence and 41 species for expression analysis with information given on methods [Sanger sequencing, whole genome sequencing, quantitative real-time polymerase chain reaction (qPCR) or retinal RNA-sequencing (transcriptomes)].

| n total | n expression |  |  |
| --- | --- | --- | --- |
| **Abudefdufinae** | | | |
| 1 (transcriptome) | 1 (transcriptome) | *Abudefduf septemfasciatus* | OK350474 |
| 2 (transcriptome) | 2 (transcriptome) | *Abudefduf sexfasciatus* | OK350479 |
| **Chrominae** | | | |
| 3 (genome) |  | *Chromis chromis* | ﻿ERX1545069 |
| 4 (Sanger) | 3 (qPCR) | *Chromis nitida* | KX766073 |
| 5 (Sanger) | 4 (qPCR) | *Chromis viridis* | HQ286500 |
| 6 (transcriptome) | 5 (transcriptome) | *Chromis weberi* | OK350529 |
| 7 (Sanger) | 6 (qPCR) | *Dascyllus aruanus* | KU745446 |
| 8 (Sanger) | 7 (qPCR) | *Dascyllus reticulatus* | KU745440 |
| 9 (transcriptome & genome) | 8 (transcriptome) | *Dascyllus trimaculatus* | OK350544 & PRJNA828170 |
| **Pomacentridae** | | | |
| 10 (transcriptome & genome) | 9 (transcriptome) | *Acanthochromis polyacanthus* | OK350485 & PRJNA888054 |
| 11 (Sanger) | 10 (qPCR) | *Amblyglyphidodon curacao* | KX766069 |
| 12 (Sanger) | 11 (qPCR) | *Amblyglyphidodon leucogaster* | KX766070 |
| 13 (Sanger) | 12 (qPCR) | *Chrysiptera brownriggii* | KX766071 |
| 14 (transcriptome) | 13 (transcriptome) | *Chrysiptera cyanea* | OK350535 |
| 15 (Sanger) |  | *Chrysiptera rex* | HQ286501 |
| 16 (Sanger) | 14 (qPCR) | *Chrysiptera rollandi* | KU745452 |
| 17 (transcriptome) | 15 (transcriptome) | *Chrysiptera starcki* | OK350539 |
| 18 (Sanger) | 16 (qPCR) | *Dischistodus perspicillatus* | KX766074 |
| 19 (Sanger) | 17 (qPCR) | *Dischistodus prosopotaenia* | KX766075 |
| 20 (Sanger) | 18 (qPCR) | *Neoglyphidodon nigroris* | KX766078 |
| 21 (Sanger) | 19 (qPCR) | *Neopomacentrus azysron* | KX766076 |
| 22 (Sanger) |  | *Neopomacentrus bankieri* | HQ286503 |
| 23 (transcriptome) | 20 (transcriptome) | *Neopomacentrus cyanomos* | OK350570 |
| 24 (Sanger) | 21 (qPCR) | *Pomacentrus adelus* | KX766079 |
| 25 (transcriptome) | 22 (transcriptome) | *Pomacentrus amboinensis* | ﻿**ON738357 (α), ON738358 (*β*)** |
| 26 (transcriptome) | 23 (transcriptome) | *Pomacentrus australis* | OK350596 (α), OK350601 (*β*) |
| 27 (Sanger) | 24 (qPCR) | *Pomacentrus chrysurus* | KX766080 |
| 28 (Sanger) | 25 (qPCR) | *Pomacentrus coelestis* | KU745434 |
| 29 (transcriptome) | 26 (transcriptome) | *Pomacentrus moluccensis* | ﻿KY458224 |
| 30 (Sanger) | 27 (qPCR) | *Pomacentrus nagasakiensis* | KU745422 |
| 31 (Sanger) | 28 (qPCR) | *Pomacentrus pavo* | KX766081 |
| 32 (Sanger) | 29 (qPCR) | *Pomacentrus wardi* | KX766082 |
| **(Amphiprionini)** | | | |
| 33 (genome) |  | *﻿**Amphiprion akallopisos* | PRJNA515163 |
| 34 (transcriptome) | 30 (transcriptome) | *Amphiprion akindynos* | MN098320 |
| 35 (genome) | 31 (transcriptome) | *Amphiprion biaculeatus* | OK350495 & **ON738359 (α), ON738360 (*β*)** |
| 36 (genome) |  | *﻿Amphiprion bicinctus* | PRJNA515163 |
| 37 (genome) |  | *Amphiprion frenatus* | SRP132439 |
| 38 (genome) | 32 (transcriptome) | *Amphiprion melanopus* | OK350501 & **ON738352 (α), ON738353 (*β*)** |
| 39 (genome) |  | *Amphiprion nigripes* | PRJNA51516 |
| 40 (genome) | 33 (transcriptome) | *Amphiprion ocellaris* | ﻿NXFZ00000000.1, PRJNA547682 |
| 41 (genome) | 34 (transcriptome) | *Amphiprion percula* | ﻿GCA_003047355.1 & OK350507 (α), OK350508 (*β*) |
| 42 (genome) | 35 (transcriptome) | *Amphiprion perideraion* | OK350513 & **ON738354 (α), ON738355 (*β*)** |
| 43 (genome) |  | *﻿Amphiprion polymnus* | PRJNA515163 |
| 44 (genome) |  | *Amphiprion sebae* | PRJNA515163 |
|  |  | **Stegastinae** |  |
| 45 (Sanger) |  | *Parma oligolepis* | HQ286504 |
| 46 (transcriptome) | 36 (transcriptome) | *Parma unifasciata* | OK350581 (α), OK350583 (*β*) |
| 47 (transcriptome) |  | *Plectroglyphidodon johnstonianus* | **ON738356 (α)** |
| 48 (transcriptome) | 37 (transcriptome) | *Plectroglyphidodon lacrymatus* | OK350588 |
| 49 (transcriptome) | 38 (transcriptome) | *Plectroglyphidodon leucozonus* | OK350594 |
| 50 (transcriptome) | 39 (transcriptome) | *Stegastes apicalis* | OK350606 |
| 51 (transcriptome) | 40 (transcriptome) | *Stegastes gascoynei* | OK350612 |
| 52 (genome) |  | *Stegastes partitus* | ﻿XM_008294175.1 |

**Table S2** Overview of sites and site effects considered for λ_max_-calculations of SWS1 in damselfish species. Site numbers refer to the corresponding bovine RH1 and to damselfish SWS1. Calculations are based on SWS1 sequence comparison to reference species with known pure protein spectral absorbance (λ_max_) **[**gained from *in-vitro* opsin protein expression studies [(*Oreochromis* *niloticus* (Parry *et al.*, 2005)], *Metriaclima* *zebra* (Spady *et al.*, 2006), and *Oryzias latipes* (Matsumoto *et al.*, 2006)], and/or to other damselfish species having a very similar sequence in known tuning sites and with known λ_max_ gained from microspectrophotomerty [*Pomacentrus amboinensis* (Siebeck *et al.*, 2010)*, P. coelestis*, and *Dascyllus trimaculatus* (McFarland & Loew, 1994)]. Only AA changes at transmembrane or retinal chromophore binding pocket regions that have previously been determined as tuning sites (Yokoyama *et al.*, 1999, 2007; Wilkie *et al.*, 2000; Hunt *et al.*, 2001; Takahashi & Ebrey, 2003; Yokoyama, 2008; Dungan *et al.*, 2016) are shown with changes in polarity (polar vs non-polar) in red. Moreover, one additional retinal binding pocket site with polarity changes that has not been described yet as tuning site is also shown.

| \|  \| **variable AA sites at known SWS1 tuning sites** \| \| \| \| \| \| \| \| \| \| \| \| \| \| **new**  **sites** \| \|  \| \| \| \| --- \| --- \| --- \| --- \| --- \| --- \| --- \| --- \| --- \| --- \| --- \| --- \| --- \| --- \| --- \| --- \| --- \| --- \| --- \| --- \| \| **SWS1bovibe** bovine RH# NP_001014890.1 \| 46 \| 49 \| 52 \| 86 \| 90 \| 91 \| 93 \| 97 \| 109 \| 113 \| 114 \| 116 \| 118 \| 265 \| 125 \|  \| \| \| Dadamselcccdamselfish AA# \| 39 \| 42 \| 45 \| 79 \| 83 \| 84 \| 86 \| 90 \| 102 \| 106 \| 107 \| 109 \| 111 \| 258 \| 118 \|  \| \| \| **Reference species with SWS1 Genbank accession numbers** \| \| \| \| \| \| \| \| \| \| \| \| \| \| \| \| **Known λ_max_ [nm]** \| \| \| *Oryzias latipes* AB223058 \| F \| F \| T \| F \| S \| V \| Q \| A \| L \| E \| A \| V \| A \| Y \| S \| 356nm \| \| \| *Oreochromis niloticus* JF262087 \| F \| F \| T \| F \| S \| V \| Q \| A \| L \| E \| A \| V \| A \| Y \| A \| 360nm \| \| \| *Maylandia zebra* JF262085 \| F \| F \| T \| F \| S \| V \| Q \| A \| L \| E \| S \| V \| S \| Y \| A \| 368nm \| \| \| *Chromis viridis* HQ286500 \| A \| F \| T \| F \| S \| V \| Q \| S \| L \| E \| S \| V \| S \| Y \| A \| 367nm \| \| \| *Dascyllus trimaculatus* JF262087 \| A \| F \| T \| F \| S \| V \| Q \| S \| M \| E \| S \| V \| S \| Y \| G \| 368nm \| \| \| *Pomacentrus amboinensis* HQ286506 \| F \| C \| T \| F \| S \| V \| Q \| A \| L \| E \| S \| V \| S \| Y \| A \| 370 nm \| \| \| *Pomacentrus coelestis* KU745434 \| F \| F \| T \| F \| S \| V \| Q \| A \| L \| E \| A \| I \| A \| Y \| A \| 360nm \| \| \| **Species to calculate λ_max_** \| \| \| \| \| \| \| \| \| \| \| \| \| \| \| \|  \| \| \|  \| \| \| \| \| \| \| \| **Abudefdufinae** \| \| \| \| \| \| \| \| **Estimated tuning effect [nm] based on reference species** \| \| \| *Abu. septemfasciatus* SWS1α \| F \| F \| T \| F \| S \| V \| Q \| A \| L \| E \| A \| V \| A \| Y \| A \| **356/360nm** = 356(*O. latipes*)/360(*P. coelestis*)/360(*O. niloticus*) \| \| \| *Abu. sexfasciatus* SWS1α \| F \| F \| T \| F \| S \| V \| Q \| A \| L \| E \| A \| V \| A \| Y \| A \| **356/360nm** = 356(*O. latipes*)/360(*P. coelestis*)/360(*O. niloticus*) \| \| \|  \| \| \| \| \| \| \| \| **Chrominae** \| \| \| \| \| \| \| \|  \| \| \| *Chr. chromis* SWS1-1  *Chr. chromis* SWS1-2 \| T  T \| F  F \| L  L \| F  F \| S  S \| V  V \| Q  Q \| C  C \| L  L \| E  E \| A  S \| V  V \| S  S \| Y  Y \| A  A \| **?** closest to 361/365nm= 356(*O. latipes*)/360(*P. coelestis*) +5(A118S) ±?T46 ±?L52  **?** closest to 368/370nm = 368(*M.* zebra)/370(*P. amboinensis*) ±?T46 ±?L5 \| \| \| *Chr. nitida* SWS1 β \| A \| C \| T \| F \| S \| V \| Q \| A \| L \| E \| A \| V \| S \| Y \| X \| **370nm** \| \| \| *Chr. viridis* SWS1β \| A \| F \| T \| F \| S \| V \| Q \| S \| L \| E \| S \| V \| S \| Y \| A \| **367nm** \| \| \| *Chr. weberi* SWS1β \| G \| F \| F \| F \| S \| V \| Q \| C \| L \| E \| S \| V \| S \| Y \| A \| **?** \| \| \| *Das. aruanus* SWS1β \| A \| F \| T \| F \| S \| V \| Q \| S \| M \| E \| S \| V \| S \| Y \| A \| **367**/**368**nm = 367(*C. viridis*)/368(*D. trimaculatus*) \| \| \| *Das. reticulatus* SWS1β \| A \| F \| T \| F \| S \| V \| Q \| S \| M \| E \| S \| V \| S \| ? \| A \| **367**/**368**nm = 367(*C. viridis*)/ 368(*D. trimaculatus*) \| \| \| *Das. trimaculatus* SWS1β \| A \| F \| T \| F \| S \| V \| Q \| S \| M \| E \| S \| V \| S \| Y \| A \| **368nm** \| \| \|  \| \| \| \| \| \| \| \| **Pomacentridae** A \| \| \| \| \| \| \| \|  \| \| \| *Aca. polyacanthus* SWS1α or β \| F \| F \| T \| F \| S \| V \| Q \| A \| L \| E \| A \| V \| S \| Y \| A \| **363/368nm** = 356(O. niloticus)/ 360(*P. coelestis*) + 2(C49) +5(A118S) \| \| \| *Amb. curacao* SWS1β \| F \| F \| T \| F \| S \| V \| Q \| A \| L \| E \| S \| V \| S \| Y \| A \| **370nm** = 370(*P. amboinensis*) \| \| \| *Amb. leucogaster* SWS1β \| X \| F \| T \| F \| S \| V \| Q \| A \| L \| E \| S \| V \| S \| Y \| A \| **368nm** = 368(*M. zebra)* \| \| \| *Chr. brownriggii* SWS1α \| F \| F \| T \| F \| S \| V \| Q \| A \| L \| E \| A \| V \| A \| Y \| G \| **356/360nm** = 356(*O. latipes*)/360(*P. coelestis*)/360(*O. niloticus*) \| \| \| *Chr. cyanea* SWS1α \| F \| F \| T \| F \| S \| V \| Q \| A \| L \| E \| A \| V \| A \| Y \| G \| **356/360nm** = 356(*O. latipes*)/360(*P. coelestis*)/360(*O. niloticus*) \| \| \| *Chr. rex* SWS1α \| F \| F \| T \| F \| S \| V \| Q \| A \| L \| E \| A \| V \| S \| Y \| G \| **361/365nm** = 356(*O. latipes*)/360(*P. coelestis*)/360(*O. niloticus*) + 5(A118S) \| \| \| *Chr. rollandi* SWS1α \| F \| F \| T \| F \| S \| V \| Q \| A \| L \| E \| A \| V \| A \| Y \| G \| **356/360nm** = 356(*O. latipes*)/360(*P. coelestis*)/360(*O. niloticus*) \| \| \| *Chr. starcki* SWS1α \| F \| F \| T \| F \| S \| V \| Q \| A \| L \| E \| A \| V \| A \| Y \| A \| **356/360nm** = 356(*O. latipes*)/360(*P. coelestis*)/360(*O. niloticus*) \| \| \| *Dis. perspicillatus* SWS1α \| F \| F \| T \| F \| S \| V \| Q \| A \| L \| E \| A \| V \| A \| Y \| G \| **356/360nm** = 356(*O. latipes*)/360(*P. coelestis*)/360(*O. niloticus*) \| \| \| *Dis. prosopotaenia* SWS1α \| F \| F \| T \| F \| S \| V \| Q \| A \| L \| E \| A \| V \| A \| Y \| A \| **356/360nm** = 356(*O. latipes*)/360(*P. coelestis*)/360(*O. niloticus*) \| \| \| *Neo. nigroris* SWS1β \| F \| L \| T \| F \| S \| V \| Q \| A \| L \| E \| S \| V \| S \| Y \| A \| **368/370nm** = 368(*M.* zebra)/370(*P. amboinensis*) \| \| \| *Neo. azysron* SWS1β \| F \| C \| T \| F \| S \| V \| Q \| A \| L \| E \| S \| V \| S \| Y \| A \| **370nm** = 370(*P. amboinensis*) \| \| \|  \| **variable AA sites at known SWS1 tuning sites** \| \| \| \| \| \| \| \| \| \| \| \| \| \|  \|  \| \| \| \| **SWS1bovibe** bovine RH# NP_001014890.1 \| 46 \| 49 \| 52 \| 86 \| 90 \| 91 \| 93 \| 97 \| 109 \| 113 \| 114 \| 116 \| 118 \| 265 \|  \|  \| \| \| Dadamselcccdamselfish AA# \| 39 \| 42 \| 45 \| 79 \| 83 \| 84 \| 86 \| 90 \| 102 \| 106 \| 107 \| 109 \| 111 \| 258 \|  \|  \| \| \| *Neo.s bankieri* SWS1β \| F \| I \| T \| F \| S \| V \| Q \| A \| L \| E \| S \| V \| S \| Y \| A \| **368/370nm** = 368(*M.* zebra)/370(*P. amboinensis*) \| \| \| *Neo. cyanomos* SWS1β \| F \| C \| T \| F \| S \| V \| Q \| A \| L \| E \| S \| V \| S \| Y \| A \| **370nm** = 370(*P. amboinensis*) \| \| \| *Pom. adelus* \| F \| X \| T \| F \| S \| V \| Q \| A \| L \| E \| X \| X \| X \| Y \| G \| **?** \| \| \| *Pom. amboinensis SWS1* α \| F \| F \| T \| F \| S \| V \| Q \| A \| L \| E \| A \| V \| A \| Y \| G \| **356/360nm** = 356(*O. latipes*)/360(*P. coelestis*)/360(*O. niloticus*) \| \| \| *Pom.s amboinensis* SWS1β \| F \| C \| T \| F \| S \| V \| Q \| A \| L \| E \| S \| V \| S \| Y \| A \| **370nm** \| \| \| *Pom. australis* SWS1α \| F \| F \| T \| F \| S \| V \| Q \| A \| L \| E \| A \| V \| A \| Y \| A \| **356/360nm** = 356(*O. latipes*)/360(*P. coelestis*)/360(*O. niloticus*) \| \| \| *Pom. australis* SWS1β \| F \| C \| T \| F \| S \| V \| Q \| A \| L \| E \| S \| V \| S \| Y \| A \| **370nm** = 370(*P. amboinensis*) \| \| \| *Pom. chrysurus* SWS1β \| F \| L \| T \| F \| S \| V \| Q \| A \| L \| E \| S \| V \| S \| Y \| G \| **370nm** = 370(*P. amboinensis*) \| \| \| *Pom. coelestis* SWS1α \| F \| F \| T \| F \| S \| V \| Q \| A \| L \| E \| A \| I \| A \| Y \| A \| **360nm** \| \| \| *Pom. moluccensis* SWS1β \| F \| C \| T \| F \| S \| V \| Q \| A \| L \| E \| S \| V \| S \| Y \| A \| **370nm** = 370(*P. amboinensis*) \| \| \| *Pom. nagasakiensis* SWS1β \| F \| C \| T \| F \| S \| V \| Q \| A \| L \| E \| ? \| V \| S \| Y \| G \| **370nm** = 370(*P. amboinensis*) \| \| \| *Pom. pavo* SWS1α \| F \| F \| T \| F \| S \| V \| Q \| A \| L \| E \| A \| I \| A \| Y \| G \| **356/360nm** = 356(*O. latipes*)/360(*P. coelestis*)/360(*O. niloticus*) \| \| \| *Pom. wardi* SWS1α \| F \| F \| T \| F \| S \| V \| Q \| A \| L \| E \| A \| V \| A \| Y \| S \| **356/360nm** = 356(*O. latipes*)/360(*P. coelestis*)/360(*O. niloticus*) \| \| \| **(Amphiprioninae)** \|  \|  \|  \|  \|  \|  \|  \|  \|  \|  \|  \|  \|  \|  \|  \|  \| \| \| *Amp. akallopisos* SWS1α  *Amp. akallopisos* SWS1β \| F  F \| F  C \| T  T \| F  F \| S  S \| V  V \| Q  Q \| A  A \| L  L \| E  E \| A  S \| V  V \| A  S \| Y  Y \| S  A \| **356/360nm** = 356(*O. latipes*)/360(*P. coelestis*)/360(*O. niloticus*)  **370nm** = 370(*P. amboinensis*) \| \| \| *Amp. akindynos* SWS1β \| F \| C \| T \| F \| S \| V \| Q \| A \| L \| E \| S \| V \| S \| Y \| A \| **370nm** = 370(*P. amboinensis*) \| \| \| *Amp. biaculeatus* SWS1α  *Amp. biaculeatus* SWS1β \| F  F \| F  F \| T  T \| F  F \| S  S \| V  V \| Q  Q \| A  A \| L  L \| E  E \| A  S \| V  V \| A  S \| Y  Y \| S  A \| **356/360nm** = 356(*O. latipes*)/360(*P. coelestis*)/360(*O. niloticus*  **368/370nm** = 368(*M.* zebra)/370(*P. amboinensis*) \| \| \| *﻿Amp. bicinctus* SWS1α  *Amp. bicinctus* SWS1β \| F  F \| F  C \| T  T \| F  F \| S  S \| V  V \| Q  Q \| A  A \| L  L \| E  E \| A  S \| V  V \| A  S \| Y  Y \| S  A \| **356/360nm** = 356(*O. latipes*)/360(*P. coelestis*)/360(*O. niloticus*)  **370nm** = 370(*P. amboinensis*) \| \| \| *Amp. frenapus* SWS1α  *Amp. frenapus* SWS1β \| F  F \| F  C \| T  T \| F  F \| S  S \| V  V \| Q  Q \| A  A \| L  L \| E  E \| A  S \| V  V \| A  S \| Y  Y \| S  A \| **356/360nm** = 356(*O. latipes*)/360(*P. coelestis*)/360(*O. niloticus*)  **370nm** = 370(*P. amboinensis*) \| \| \| *Amp. nigripes* SWS1α  *Amp. nigripes* SWS1β \| F  F \| F  F \| T  T \| F  F \| S  S \| I  V \| Q  Q \| A  A \| L  L \| E  E \| A  S \| V  V \| A  S \| Y  Y \| S  A \| **356/360nm** = 356(*O. latipes*)/360(*P. coelestis*)/360(*O. niloticus)*  **368/370nm** = 368(*M.* zebra)/370(*P. amboinensis*) \| \| \| *Amp. melanopus* SWS1α  *Amp. melanopus* SWS1β \| F  F \| F  C \| T  T \| F  F \| S  S \| V  V \| Q  Q \| A  A \| L  L \| E  E \| A  S \| V  V \| A  S \| Y  Y \| S  A \| **356/360nm** = 356(*O. latipes*)/360(*P. coelestis*)/360(*O. niloticus*)  **370nm** = 370(*P. amboinensis*) \| \| \| *Amp. ocellaris* SWS1α  *Amp. ocellaris* SWS1β \| F  F \| F  C \| T  T \| F  F \| S  S \| V  V \| Q  Q \| A  A \| L  L \| E  E \| A  S \| V  V \| A  S \| Y  Y \| S  A \| **356/360nm** = 356(*O. latipes*)/360(*P. coelestis*)/360(*O. niloticus*)  **370nm** = 370(*P. amboinensis*) \| \| \| *Amp. percula* SWS1α  *Amp. percula* SWS1 β \| F  F \| F  C \| T  T \| F  F \| S  S \| V  V \| Q  Q \| A  A \| L  L \| E  E \| A  S \| V  V \| A  S \| Y  Y \| A  A \| **356/360nm** = 356(*O. latipes*)/360(*P. coelestis*)/360(*O. niloticus*)  **370nm** = 370(*P. amboinensis*) \| \| \| *Amp. perideraion* SWS1α  *Amp. perideraion* SWS1β \| F  F \| F  C \| T  T \| F  F \| S  S \| V  V \| Q  Q \| A  A \| L  L \| E  E \| A  S \| V  V \| A  S \| Y  Y \| S  A \| **356/360nm** = 356(*O. latipes*)/360(*P. coelestis*)/360(*O. niloticus*)  **370nm** = 370(*P. amboinensis*) \| \| \| *﻿Amp. polymnus* SWS1α  *Amp. polymnus* SWS1β \| F  F \| F  F \| T  T \| F  F \| S  S \| V  V \| Q  Q \| A  A \| L  L \| E  E \| A  S \| V  V \| A  S \| Y  Y \| S  A \| **356/360nm** = 356(*O. latipes*)/360(*P. coelestis*)/360(*O. niloticus)*  **368nm** = 368(*M.* zebra) \| \| \| *Amp. sebae* SWS1α  *Amp. sebae* SWS1β \| F  F \| C  C \| T  T \| F  F \| S  S \| V  V \| Q  Q \| A  A \| L  L \| E  E \| A  S \| V  V \| A  S \| Y  Y \| S  A \| **358/362nm** = 356(O. niloticus)/ 360(*P. coelestis*) + 2(C49)  **370nm** = 370(*P. amboinensis*) \| \| \| **Stegastinae** \|  \|  \|  \|  \|  \|  \|  \|  \|  \|  \|  \|  \|  \|  \|  \|  \| \| \| *Par. oligolepis* SWS1α \| I \| F \| T \| F \| S \| V \| Q \| A \| L \| E \| A \| V \| A \| Y \| S \| **356/360nm** = 356(*O. latipes*)/360(*P. coelestis*)/360(*O. niloticus*) \| \| \| *Par. unifasciata* SWS1αx1  *Par. unifasciata* SWS1x2 \| F  F \| F  F \| T  T \| F  F \| S  S \| V  V \| Q  Q \| A  A \| L  L \| E  E \| A  A \| V  V \| A  A \| Y  Y \| S  S \| **356/360nm** = 356(*O. latipes*)/360(*P. coelestis*)/360(*O. niloticus*)  **356/360nm** = 356(*O. latipes*)/360(*P. coelestis*)/360(*O. niloticus*) \| \| \| *Ste. apicalis* SWS1α \| F \| F \| T \| F \| S \| V \| Q \| A \| L \| E \| A \| V \| A \| Y \| S \| **356/360nm** = 356(*O. latipes*)/360(*P. coelestis*)/360(*O. niloticus*) \| \| \| *Ste. gasconynei* SWS1α \| F \| F \| T \| F \| S \| V \| Q \| A \| L \| E \| A \| V \| A \| Y \| A \| **356/360nm** = 356(*O. latipes*)/360(*P. coelestis*)/360(*O. niloticus*) \| \| \| *Ste. partitus* SWS1α \| F \| F \| T \| F \| S \| V \| Q \| A \| L \| E \| A \| V \| A \| Y \| S \| **356/360nm** = 356(*O. latipes*)/360(*P. coelestis*)/360(*O. niloticus*) \| \| \| *Ple. dickii* SWS1α \| F \| F \| T \| F \| S \| V \| Q \| A \| L \| E \| A \| V \| A \| Y \| S \| **356/360nm** = 356(*O. latipes*)/360(*P. coelestis*)/360(*O. niloticus*) \| \| \| *Ple. johnstonianus* SWS1α \| F \| F \| T \| F \| S \| V \| Q \| A \| L \| E \| A \| V \| A \| Y \| S \| **356/360nm** = 356(*O. latipes*)/360(*P. coelestis*)/360(*O. niloticus*) \| \| \| *Ple. lacrymatus* SWS1α \| I \| F \| T \| F \| S \| V \| Q \| A \| L \| E \| A \| V \| A \| Y \| S \| **356/360nm** = 356(*O. latipes*)/360(*P. coelestis*)/360(*O. niloticus*) \| \| |
| --- | --- | --- | --- | --- | --- | --- | --- | --- | --- | --- | --- | --- | --- | --- | --- | --- | --- | --- | --- | --- | --- | --- | --- | --- | --- | --- | --- | --- | --- | --- | --- | --- | --- | --- | --- | --- | --- | --- | --- | --- | --- | --- | --- | --- | --- | --- | --- | --- | --- | --- | --- | --- | --- | --- | --- | --- | --- | --- | --- | --- | --- | --- | --- | --- | --- | --- | --- | --- | --- | --- | --- | --- | --- | --- | --- | --- | --- | --- | --- | --- | --- | --- | --- | --- | --- | --- | --- | --- | --- | --- | --- | --- | --- | --- | --- | --- | --- | --- | --- | --- | --- | --- | --- | --- | --- | --- | --- | --- | --- | --- | --- | --- | --- | --- | --- | --- | --- | --- | --- | --- | --- | --- | --- | --- | --- | --- | --- | --- | --- | --- | --- | --- | --- | --- | --- | --- | --- | --- | --- | --- | --- | --- | --- | --- | --- | --- | --- | --- | --- | --- | --- | --- | --- | --- | --- | --- | --- | --- | --- | --- | --- | --- | --- | --- | --- | --- | --- | --- | --- | --- | --- | --- | --- | --- | --- | --- | --- | --- | --- | --- | --- | --- | --- | --- | --- | --- | --- | --- | --- | --- | --- | --- | --- | --- | --- | --- | --- | --- | --- | --- | --- | --- | --- | --- | --- | --- | --- | --- | --- | --- | --- | --- | --- | --- | --- | --- | --- | --- | --- | --- | --- | --- | --- | --- | --- | --- | --- | --- | --- | --- | --- | --- | --- | --- | --- | --- | --- | --- | --- | --- | --- | --- | --- | --- | --- | --- | --- | --- | --- | --- | --- | --- | --- | --- | --- | --- | --- | --- | --- | --- | --- | --- | --- | --- | --- | --- | --- | --- | --- | --- | --- | --- | --- | --- | --- | --- | --- | --- | --- | --- | --- | --- | --- | --- | --- | --- | --- | --- | --- | --- | --- | --- | --- | --- | --- | --- | --- | --- | --- | --- | --- | --- | --- | --- | --- | --- | --- | --- | --- | --- | --- | --- | --- | --- | --- | --- | --- | --- | --- | --- | --- | --- | --- | --- | --- | --- | --- | --- | --- | --- | --- | --- | --- | --- | --- | --- | --- | --- | --- | --- | --- | --- | --- | --- | --- | --- | --- | --- | --- | --- | --- | --- | --- | --- | --- | --- | --- | --- | --- | --- | --- | --- | --- | --- | --- | --- | --- | --- | --- | --- | --- | --- | --- | --- | --- | --- | --- | --- | --- | --- | --- | --- | --- | --- | --- | --- | --- | --- | --- | --- | --- | --- | --- | --- | --- | --- | --- | --- | --- | --- | --- | --- | --- | --- | --- | --- | --- | --- | --- | --- | --- | --- | --- | --- | --- | --- | --- | --- | --- | --- | --- | --- | --- | --- | --- | --- | --- | --- | --- | --- | --- | --- | --- | --- | --- | --- | --- | --- | --- | --- | --- | --- | --- | --- | --- | --- | --- | --- | --- | --- | --- | --- | --- | --- | --- | --- | --- | --- | --- | --- | --- | --- | --- | --- | --- | --- | --- | --- | --- | --- | --- | --- | --- | --- | --- | --- | --- | --- | --- | --- | --- | --- | --- | --- | --- | --- | --- | --- | --- | --- | --- | --- | --- | --- | --- | --- | --- | --- | --- | --- | --- | --- | --- | --- | --- | --- | --- | --- | --- | --- | --- | --- | --- | --- | --- | --- | --- | --- | --- | --- | --- | --- | --- | --- | --- | --- | --- | --- | --- | --- | --- | --- | --- | --- | --- | --- | --- | --- | --- | --- | --- | --- | --- | --- | --- | --- | --- | --- | --- | --- | --- | --- | --- | --- | --- | --- | --- | --- | --- | --- | --- | --- | --- | --- | --- | --- | --- | --- | --- | --- | --- | --- | --- | --- | --- | --- | --- | --- | --- | --- | --- | --- | --- | --- | --- | --- | --- | --- | --- | --- | --- | --- | --- | --- | --- | --- | --- | --- | --- | --- | --- | --- | --- | --- | --- | --- | --- | --- | --- | --- | --- | --- | --- | --- | --- | --- | --- | --- | --- | --- | --- | --- | --- | --- | --- | --- | --- | --- | --- | --- | --- | --- | --- | --- | --- | --- | --- | --- | --- | --- | --- | --- | --- | --- | --- | --- | --- | --- | --- | --- | --- | --- | --- | --- | --- | --- | --- | --- | --- | --- | --- | --- | --- | --- | --- | --- | --- | --- | --- | --- | --- | --- | --- | --- | --- | --- | --- | --- | --- | --- | --- | --- | --- | --- | --- | --- | --- | --- | --- | --- | --- | --- | --- | --- | --- | --- | --- | --- | --- | --- | --- | --- | --- | --- | --- | --- | --- | --- | --- | --- | --- | --- | --- | --- | --- | --- | --- | --- | --- | --- | --- | --- | --- | --- | --- | --- | --- | --- | --- | --- | --- | --- | --- | --- | --- | --- | --- | --- | --- | --- | --- | --- | --- | --- | --- | --- | --- | --- | --- | --- | --- | --- | --- | --- | --- | --- | --- | --- | --- | --- | --- | --- | --- | --- | --- | --- | --- | --- | --- | --- | --- | --- | --- | --- | --- | --- | --- | --- | --- | --- | --- | --- | --- | --- | --- | --- | --- | --- | --- | --- | --- | --- | --- | --- | --- | --- | --- | --- | --- | --- | --- | --- | --- | --- | --- | --- | --- | --- | --- | --- | --- | --- | --- | --- | --- | --- | --- | --- | --- | --- | --- | --- | --- | --- | --- | --- | --- | --- | --- | --- | --- | --- | --- | --- | --- | --- | --- | --- | --- | --- | --- | --- | --- | --- | --- | --- | --- | --- | --- | --- | --- | --- | --- | --- | --- | --- | --- | --- | --- | --- | --- | --- | --- | --- | --- | --- | --- | --- | --- | --- | --- | --- | --- | --- | --- | --- | --- | --- | --- | --- | --- | --- | --- | --- | --- | --- | --- | --- | --- | --- | --- | --- | --- | --- | --- | --- | --- | --- | --- | --- | --- | --- | --- | --- | --- | --- | --- | --- | --- | --- | --- | --- | --- | --- | --- | --- | --- | --- | --- | --- | --- | --- | --- | --- | --- | --- | --- | --- | --- | --- | --- | --- | --- | --- | --- | --- | --- | --- | --- | --- | --- | --- | --- | --- | --- | --- | --- | --- | --- | --- | --- | --- | --- | --- | --- | --- | --- | --- | --- | --- | --- | --- | --- | --- | --- | --- | --- | --- | --- | --- | --- | --- | --- | --- | --- | --- | --- | --- | --- | --- | --- | --- | --- | --- | --- | --- | --- | --- | --- | --- | --- | --- | --- | --- | --- | --- | --- | --- | --- | --- | --- | --- | --- | --- | --- | --- | --- | --- | --- | --- | --- | --- | --- | --- | --- | --- | --- | --- | --- | --- | --- | --- | --- | --- | --- | --- | --- | --- | --- | --- | --- | --- | --- | --- | --- | --- | --- | --- | --- | --- | --- | --- | --- | --- | --- | --- | --- | --- | --- | --- | --- | --- | --- | --- | --- | --- | --- | --- | --- | --- | --- | --- | --- | --- | --- | --- | --- | --- | --- | --- | --- | --- | --- | --- | --- | --- | --- | --- | --- | --- | --- | --- | --- | --- | --- | --- | --- | --- | --- | --- | --- | --- | --- | --- | --- | --- | --- | --- | --- | --- | --- | --- | --- | --- | --- | --- | --- | --- | --- | --- | --- | --- | --- | --- | --- | --- | --- | --- | --- | --- | --- | --- | --- | --- | --- | --- | --- | --- | --- | --- | --- | --- | --- | --- | --- | --- | --- | --- | --- | --- | --- | --- | --- | --- | --- | --- | --- | --- | --- | --- | --- | --- | --- | --- | --- | --- | --- | --- | --- | --- | --- | --- | --- | --- | --- | --- | --- | --- | --- | --- | --- | --- | --- | --- | --- | --- | --- | --- | --- | --- | --- | --- | --- | --- | --- | --- | --- | --- | --- | --- | --- | --- | --- | --- | --- | --- | --- | --- | --- | --- | --- | --- | --- | --- | --- | --- | --- | --- | --- | --- | --- | --- | --- | --- | --- | --- | --- | --- | --- | --- | --- | --- | --- | --- | --- | --- | --- | --- | --- | --- | --- | --- | --- | --- | --- | --- | --- | --- | --- | --- | --- | --- | --- | --- | --- | --- | --- | --- | --- | --- | --- | --- | --- | --- | --- | --- | --- | --- | --- | --- | --- | --- | --- | --- | --- | --- | --- | --- | --- | --- | --- | --- | --- | --- | --- | --- | --- | --- | --- | --- | --- | --- | --- | --- | --- | --- | --- | --- | --- | --- | --- | --- | --- | --- | --- | --- | --- | --- | --- | --- | --- | --- | --- | --- | --- | --- | --- | --- | --- | --- | --- | --- | --- | --- | --- | --- | --- | --- | --- | --- | --- | --- | --- | --- | --- | --- | --- | --- | --- | --- | --- | --- | --- | --- | --- |

**Table S3:** Summary of damselfish species split by genera including the number of sampled species, i.e. species with genomic marker information according to McCord *et al.* (2021), the number of total species according to Eschmeyer`s Catalog of Fishes (Fricke *et al.*, 2023), and the number of missing and fraction of underrepresented species used for BAMM analysis.

| **genera** | **# sampled species** | **# of total extant species** | **# missing species** | **% underrepresentation** |
| --- | --- | --- | --- | --- |
| Abudefduf | 21 | 21 | 0 | 0 |
| Acanthochromis | 1 | 1 | 0 | 0 |
| Altrichthys | 3 | 3 | 0 | 0 |
| Amblyglyphidodon | 11 | 11 | 0 | 0 |
| Amblypomacentrus | 5 | 5 | 0 | 0 |
| Amphiprion | 28 | 30 | 2 | 7 |
| Azurina | 7 | 10 | 3 | 30 |
| Cheiloprion | 1 | 1 | 0 | 0 |
| Chromis + Pycnochromis | 81 | 109 | 28 | 26 |
| Chrysiptera | 29 | 40 | 11 | 28 |
| Dascyllus | 10 | 11 | 1 | 9 |
| Dischistodus | 5 | 7 | 2 | 29 |
| Hemiglyphidodon | 1 | 1 | 0 | 0 |
| Hypsypops | 1 | 1 | 0 | 0 |
| Lepidozygus | 1 | 1 | 0 | 0 |
| Mecaenichthys | 1 | 1 | 0 | 0 |
| Microspathodon | 3 | 4 | 1 | 25 |
| Neoglyphidodon | 7 | 9 | 2 | 22 |
| Neopomacentrus | 16 | 16 | 0 | 0 |
| Nexilosus | 1 | 1 | 0 | 0 |
| Parma | 6 | 10 | 4 | 40 |
| Plectroglyphidodon | 17 | 19 | 2 | 11 |
| Pomacentrus | 57 | 82 | 25 | 30 |
| Pomachromis | 2 | 4 | 2 | 50 |
| Pristotis | 1 | 2 | 1 | 50 |
| Similiparma | 2 | 2 | 0 | 0 |
| Stegastes | 26 | 39 | 13 | 33 |
| Teixeirichthys | 1 | 1 | 0 | 0 |

**Table S4:** Summary of damselfish proportional single cone opsin expression, and lens transmission (T50 = wavelength of 50% transmission). ^*^this study, ^1^(Stieb *et al.*, 2016), ^2^(Stieb *et al.*, 2017), ^3^(Luehrmann *et al.*, 2018), ^4^(Stieb *et al.*, 2019), ^5^(Stieb *et al.*, 2023), ^6^(Mitchell *et al.*, 2021), ^7^(Siebeck & Marshall, 2007).

|  | n | **SWS1 (short, α)** | **SWS1 (long, β)** | **SWS2B** | Lens T50 |
| --- | --- | --- | --- | --- | --- |
| **Abudefdufinae** |  |  |  |  |  |
| *Abudefduf septemfasciatus* | 2^5^ | 100 | 0 | 0 | 340 |
| *Abudefduf sexfasciatus* | 2^5^ | 99.5 ± 0.6 | 0 | 0.5 ± 0.6 | 350 |
| **Chrominae** |  |  |  |  |  |
| *Chromis nitida* | 6^1^ | 0 | 54.0 ± 10.7 | 46.0 ± 10.7 | 361-64^1,7^ |
| *Chromis viridis* | 8^1^ | 0 | 99.6 ± 0.4 | 0.4 ± 0.4 | 336_1_ |
| *Chromis weberi* | 1^5^ | 0 | 13.3 | 86.7 | 366 |
| *Dascyllus aruanus* | 19^2^ | 0 | 96.7 ± 5.7 | 3.3 ± 5.7 | 328^1,7^ |
| *Dascyllus reticulatus* | 13^2^ | 0 | 99.9 ± 0.2 | 0.1 ± 0.2 | 345^7^/55^1^ |
| *Dascyllus trimaculatus* | 1^5^ | 0 | 99.6 | 0.4 | 356 |
| **Pomacentrinae** |  |  |  |  |  |
| *Acanthochromis polyacanthus* | 6^1^; 1 | 99.8 ± 0; 97.6 | 0; 0 | 0.2 ± 0; 2.4 | 350^1^ |
| *Amblyglyphidodon curacao* | 8^1^ | 0 | 100 | 0 | 340-50^1^ |
| *Amblyglyphidodon leucogaster* | 6^1^ | 0 | 100 | 0 | 350^7^ |
| *Chrysiptera brownriggii* | 4^1^ | 94.3 ± 6 | 0 | 5.7 ± 6 | N/A |
| *Chrysiptera cyanea* | 8^1^; 1 | 100 ± 0.1; 100 | 0; 0 | 0.1 ± 0.1; 0 | 326-39^1,7^ |
| *Chrysiptera rollandi* | 14^2^ | 99.8 ± 0.4 | 0 | 0.2 ± 0.4 | 348^7^ |
| *Chrysiptera starcki* | 2^5^ | 100 | 0 | 0 | 341 |
| *Dischistodus perspicillatus* | 4^1^ | 99.8 ± 0.4 | 0 | 0.2 ± 0.4 | 355^7^ |
| *Dischistodus prosopotaenia* | 6^1^ | 99.7 ± 0.4 | 0 | 0.3 ± 0.4 | 350^7^ |
| *Neopomacentrus azysron* | 2^1^ | 0 | 99.9 ± 0.1 | 0.1 ± 0.1 | 324^7^/35^1^ |
| *Neopomacentrus cyanomos* | 5^1^; 1 | 0; 0 | 40.4 ± 8.8; 47.0 | 59.6 ± 8.8; 53.0 | 362^1^ |
| *Neoglyphidodon nigroris* | 2^1^ | 0 | 85.8 ± 0.7 | 14.2 ± 0.7 | N/A |
| *Pomacentrus adelus* | 2^1^ | 100 (?: α or β) | 0 | 0 | N/A |
| *Pomacentrus amboinensis* | 21^2^; 1 | 99.8 ± 0.5; 19.8 | 0; 72.0 | 0.2 ± 0.5; 8.2 | 345^1^ |
| *Pomacentrus australis* | 1^5^ | 11.3 | 85.3 | 3.4 | 347 |
| *Pomacentrus chrysurus* | 5^1^ | 0 | 99.2 ± 0.8 | 0.8 ± 0.8 | 339^1^ |
| *Pomacentrus coelestis* | 15^2^ | 100 | 0 | 0 | 339^7^/44^7^ |
| *Pomacentrus moluccensis* | 14^2^; 1 | 0; 0 | 99.9 ± 0.3; 99.0 | 0.1 ± 0.3; 1.0 | 349-73^1,7^ |
| *Pomacentrus nagasakiensis* | 14^2^ | 0 | 99.5 ± 0.9 | 0.5 ± 0.9 | 341^1^ |
| *Pomacentrus pavo* | 8^1^ | 100 | 0 | 0 | 350^1^ |
| *Pomacentrus wardi* | 2^1^ | 99.7 ± 0.4 | 0 | 0.3 ± 0.4 | 340^7^ |
| ***(Amphiprioninae)*** |  |  |  |  |  |
| *Amphiprion akindynos* | 10^4^ | 0 | 89.3 ± 10.7 | 10.7 ± 10.7 | 361^7^/343^1^ |
| *female* | 4 | 0 | 94.5 ± 5.8 | 5.5 ± 5.8 |  |
| *male* | 2 | 0 | 85.0 ± 15.8 | 15.0 ± 15.8 |  |
| *juvenil* | 4 | 0 | 86.1 ± 12.9 | 13.9 ± 12.9 |  |
| *Amphiprion biaculeatus* | 6^5,*^ | 6.5 ± 1.7 | 93.3 ± 1.7 | 0.2 ± 0.2 | 350^7^ |
| *female* | 3 | 6.1 ± 2.5 | 93.8 ± 2.5 | 0.1 ± 0.1 |  |
| *male* | 2 | 7.2 ± 0.9 | 92.6 ± 0.7 | 0.3 ± 0.2 |  |
| *juvenil* | 1 | 6.3 | 93.2 | 0.6 |  |
| *Amphiprion melanopus* | 10^5,*^ | 11.8 ± 14.1 | 77.3 ± 14.1 | 10.9 ± 11.6 | 361 |
| *female* | 4 | 7.0 ± 4.3 | 82.1 ± 7.2 | 10.9 ± 10.5 |  |
| *male* | 4 | 17.1 ± 21.1 | 68.6 ± 16.6 | 14.3 ± 13.7 |  |
| *juvenil* | 2 | 6.9 ± 4.1 | 89.3 ± 1.0 | 3.9 ± 5.1 |  |
| *Amphiprion ocellaris* | 4^6^ | 0 | 59.1 ± 9.4 | 40.8 ± 8.7 | N/A |
| *female* | 2 | 0.8 ± 1.1 | 58.7 ± 15.1 | 40.6 ± 14.1 |  |
| *male* | 2 | 0 | 59.5 ± 5.9 | 40.5 ± 5.9 |  |
| *Amphiprion percula* | 4^5,*^ | 24.4 ± 12.6 | 75.6 ± 12.6 | 0 | 320 |
| *female* | 2 | 23.2 ± 7.5 | 76.8 ± 7.5 | 0 |  |
| *male* | 1 | 39.9 | 60.1 | 0 |  |
| *juvenil* | 1 | 11.2 | 88.8 | 0 |  |
| *Amphiprion perideraion* | 4^5,*^ | 5.6 ± 1.0 | 84.5 ± 9.6 | 9.9 ± 9.9 | 380^7^ |
| *female* | 1 | 4.6 | 79.6 | 15.8 |  |
| *male* | 2 | 5.4 ± 0.3 | 84.0 ± 14.7 | 10.5 ± 14.5 |  |
| *juvenil* | 1 | 6.9 | 90.4 | 2.7 |  |
| **Stegastinae** |  |  |  |  |  |
| *Stegastes apicalis* | 1^5^ | 99.7 | 0 | 0.3 | 349-64^1,7^ |
| *Stegastes gascoynei* | 1^5^ | 57.6 | 0 | 42.4 | 349 |
| *Stegastes partitus* | N/A | | | | 365 |
| *Parma unifasciata* | 1^5^ | 44.8 (x1) 39.0 (x2) | 0 | 16.2 | 340 |
| *Plectroglyphidodon lacrymatus* | 2^5^ | 57.8 ± 16.3 | 0 | 42.2 ± 16.3 | N/A |
| *Plectroglyphidodon leucozonus* | 1^5^ | 93.4 | 0 | 6.6 | 335 |

**Figure S1:** Diversification rates throughout the history of Pomacentridae (using the phylogenetic tree of McCord *et al.* (2021) highlighting that elevated rates are only found within a subclade of Amphiprionini. The main duplication event of SWS1 (species having the main SWS1 duplication are indicated with an orange dot at the tip) is likely to have occurred within the Pomacentrinae radiation at the split between the clade composed of Pomacentrinae 1 (Chrysiptera, Dischistodus, Pomachromis, Cheiliprion) and 2 (Hemiglyphidodon*,* Amblyglyphidon*,* Acanthochromis, Altrichthys*,* Neoglyphidodon*)* and the clade composed of 3 (Pristotis*,*Teixeirichthys and Neopomacentrus), 4 (Amphiprionini), and 5 (Pomacentrus*,* Amblypomacentrus). The minor duplication event is so far only reported for *Chromis Chromis* (indicated by a red dot at the tip).

References

Dungan, S.Z., Kosyakov, A. & Chang, B.S.W. 2016. Spectral tuning of killer whale (*Orcinus orca*) rhodopsin: Evidence for positive selection and functional adaptation in a cetacean visual pigment. *Mol. Biol. Evol.* 33: 323–336. Oxford University Press.

Fricke, R., Eschmeyer, W. N. & Van der Laan, R. (eds) 2023.  Eschmeyer`s Catalog of Fishes: Genera, Species, References. Electronic version accessed dd mmm 2023.

(<http://researcharchive.calacademy.org/research/ichthyology/catalog/fishcatmain.asp>).

Hunt, D.M., Dulai, K.S., Partridge, J.C., Cottrill, P. & Bowmaker, J.K. 2001. The molecular basis for spectral tuning of rod visual pigments in deep-sea fish. *J. Exp. Biol.* 204: 3333–44.

Luehrmann, M., Stieb, S.M., Carleton, K.L., Pietzker, A., Cheney, K.L. & Marshall, N.J. 2018. Short-term colour vision plasticity on the reef: changes in opsin expression under varying light conditions differ between ecologically distinct fish species. *J. Exp. Biol.* 221: jeb175281.

Matsumoto, Y., Fukamachi, S., Mitani, H. & Kawamura, S. 2006. Functional characterization of visual opsin repertoire in Medaka (Oryzias latipes). *Gene* 371: 268–278.

McCord, C.L., Nash, C.M., Cooper, W.J. & Westneat, M.W. 2021. Phylogeny of the damselfishes (Pomacentridae) and patterns of asymmetrical diversification in body size and feeding ecology. *PLoS One* 16: 1–30.

McFarland, W.N. & Loew, E.R. 1994. Ultraviolet visual pigments in marine fishes of the family pomacentridae. *Vision Res.* 34: 1393–6.

Mitchell, L.J., Cheney, K.L., Lührmann, M., Marshall, J., Michie, K. & Cortesi, F. 2021. Molecular Evolution of Ultraviolet Visual Opsins and Spectral Tuning of Photoreceptors in Anemonefishes (Amphiprioninae). *Genome Biol. Evol.* 13: 1–14.

Parry, J.W.L., Carleton, K.L., Spady, T., Carboo, A., Hunt, D.M. & Bowmaker, J.K. 2005. Mix and match color vision: tuning spectral sensitivity by differential opsin gene expression in Lake Malawi cichlids. *Curr. Biol.* 15: 1734–9.

Siebeck, U.E. & Marshall, N.J. 2007. Potential ultraviolet vision in pre-settlement larvae and settled reef fish--a comparison across 23 families. *Vision Res.* 47: 2337–52.

Siebeck, U.E., Parker, A.N., Sprenger, D., Mäthger, L.M. & Wallis, G. 2010. A Species of Reef Fish that Uses Ultraviolet Patterns for Covert Face Recognition. *Curr. Biol.* 20: 407–410.

Spady, T.C., Parry, J.W.L., Robinson, P.R., Hunt, D.M., Bowmaker, J.K. & Carleton, K.L. 2006. Evolution of the cichlid visual palette through ontogenetic subfunctionalization of the opsin gene arrays. *Mol. Biol. Evol.* 23: 1538–47.

Stieb, S.M., Carleton, K.L., Cortesi, F., Marshall, N.J. & Salzburger, W. 2016. Depth dependent plasticity in opsin gene expression varies between damselfish (Pomacentridae) species. *Mol. Ecol.* 25: 3645–3661.

Stieb, S.M., Cortesi, F., Sueess, L., Carleton, K.L., Salzberger, W. & Marshall, N.J. 2017. Why UV- and red-vision are important for damselfish (Pomacentridae): Structural and expression variation in opsin genes. *Mol. Ecol.* 26: 1323–1342.

Stieb, S.M., de Busserolles, F., Carleton, K.L., Cortesi, F., Chung, W.S., Dalton, B.E., *et al.* 2019. A detailed investigation of the visual system and visual ecology of the Barrier Reef anemonefish, Amphiprion akindynos. *Sci. Rep.* 9: 1–14.

Stieb, S.M., Carleton, K.L., Seehausen, O., Cortesi, F. & Marshall, N. 2023. Long-wavelength-sensitive opsin expression, foraging and visual communication in coral reef fishes. *Mol. Ecol.*

Takahashi, Y. & Ebrey, T.G. 2003. Molecular basis of spectral tuning in the newt short wavelength sensitive visual pigment. *Biochemistry* 42: 6025–6034.

Wilkie, S.E., Robinson, P.R., Cronin, T.W., Poopalasundaram, S., Bowmaker, J.K. & Hunt, D.M. 2000. Spectral tuning of avian violet- and ultraviolet-sensitive visual pigments. *Biochemistry* 39: 7895–7901.

Yokoyama, S. 2008. Evolution of dim-light and color vision pigments. *Annu. Rev. Genomics Hum. Genet.* 9: 259–82.

Yokoyama, S., Takenaka, N. & Blow, N. 2007. A novel spectral tuning in the short wavelength-sensitive (SWS1 and SWS2) pigments of bluefin killifish (Lucania goodei). *Gene* 396: 196–202.

Yokoyama, S., Zhang, H., Radlwimmer, F.B. & Blow, N.S. 1999. Adaptive evolution of color vision of the Comoran coelacanth (Latimeria chalumnae). *Proc. Natl. Acad. Sci.* 96: 6279–6284.
